# A combined case-control and molecular source attribution study of human *Campylobacter* infections in Germany, 2011–2014

**DOI:** 10.1038/s41598-017-05227-x

**Published:** 2017-07-11

**Authors:** Bettina M. Rosner, Anika Schielke, Xavier Didelot, Friederike Kops, Janina Breidenbach, Niklas Willrich, Greta Gölz, Thomas Alter, Kerstin Stingl, Christine Josenhans, Sebastian Suerbaum, Klaus Stark

**Affiliations:** 1 Department of Infectious Disease Epidemiology, Robert Koch Institute, Berlin, Germany; 2 Department of Infectious Disease Epidemiology, Imperial College London, London, UK; 3 Institute for Medical Microbiology and Hospital Epidemiology, Hannover Medical School, Hannover, Germany; 4 DZIF-German Centre for Infection Research, Hannover-Braunschweig Site, Hannover, Germany; 5 Institute of Food Safety and Food Hygiene, Centre for Veterinary Public Health, Freie Universität Berlin, Berlin, Germany; 6 National Reference Laboratory for Campylobacter, Federal Institute for Risk Assessment, Berlin, Germany; 7 Max von Pettenkofer Institute, Ludwig-Maximilians-Universität München, München, Germany

## Abstract

*Campylobacter* infection is the most commonly notified bacterial enteritis in Germany. We performed a large combined case-control and source attribution study (Nov 2011-Feb 2014) to identify risk factors for sporadic intestinal *Campylobacter* infections and to determine the relative importance of various animal sources for human infections in Germany. We conducted multivariable logistic regression analysis to identify risk factors. Source attribution analysis was performed using the asymmetric island model based on MLST data of human and animal/food isolates. As animal sources we considered chicken, pig, pet dog or cat, cattle, and poultry other than chicken. Consumption of chicken meat and eating out were the most important risk factors for *Campylobacter* infections. Additional risk factors were preparation of poultry meat in the household; preparation of uncooked food and raw meat at the same time; contact with poultry animals; and the use of gastric acid inhibitors. The mean probability of human *C. jejuni* isolates to originate from chickens was highest (74%), whereas pigs were a negligible source for *C. jejuni* infections. Human *C. coli* isolates were likely to originate from chickens (56%) or from pigs (32%). Efforts need to be intensified along the food chain to reduce *Campylobacter* load, especially on chicken meat.

## Introduction

Intestinal *Campylobacter* infections are the most frequently reported bacterial infections in Germany and in other European countries^1, 2^. An overall increasing trend has been observed in Germany, from 55,000 laboratory-diagnosed *Campylobacter* infections reported in 2001 to 70,190 reported in 2015 (87 infections/100,000 population)^1^. Most intestinal *Campylobacter* infections are caused by *Campylobacter jejuni* (90%) and *Campylobacter coli* (7%), and are acquired in Germany (92%)^3^. Typical symptoms are diarrhoea, abdominal pain and fever. Sequelae such as reactive arthritis, irritable bowel syndrome, and neurological complications such as Guillain Barré syndrome can also occur, albeit with lower incidence^4–6^. The vast majority of cases (97%) are reported as sporadic, that is not as part of an outbreak^3^. Campylobacteriosis is a zoonotic disease. Important animal reservoirs for the organism are poultry, in particular chicken, and cattle^7–14^. Humans typically become infected via consumption of meat. Chicken meat plays an important role, because it is often contaminated with *Campylobacter* ^15–17^. Epidemiological studies conducted in several European and non-European countries have identified the consumption of poultry or chicken meat as an important risk factor for campylobacteriosis^14, 18–23^, and in source attribution studies outside of Germany about 50–90% of human infections were attributed to chicken^7, 8, 10, 11, 13, 14, 24^. Few studies have combined epidemiological and source attribution data^10, 14, 25^. The aim of our study was to identify risk factors for sporadic *Campylobacter* infections in Germany and combine epidemiological, molecular typing and source attribution data to determine the relative importance of potential sources for human infections.

## Methods

The study was approved by the German data protection authority, Bonn, Germany (Number III-401/008#0045; 28 July 2011), and by the ethical committee of the Charité University Medicine Berlin, Germany (Number EA2/012/11; 14 March 2011). The study was conducted in accordance with relevant guidelines and regulations. Informed consent was obtained from all subjects.

### Data availability

Data are available in Supplementary Information. Additional data of the case-control study will be made available upon request to the corresponding first or the last author.

### Study design

The study was conducted between 1 November 2011 and 28 February 2014 in rural county districts of the federal state of Brandenburg and in Berlin (urban region). To increase the number of study participants under the age of 15 years, the study region for this age group was expanded in 2013 to select urban and rural county districts in North Rhine-Westphalia and Saxony. Regions were classified as rural or urban as described previously^3^. According to the German Protection against Infection Act of 2001, laboratory diagnosed *Campylobacter* infections in patients have to be reported to the local health authority by the primary diagnostic laboratories. Local health authorities contacted patients to obtain informed consent for study participation. Patients willing to participate were sent a self-administered questionnaire by the local health authority, which was mailed free of charge directly to the Robert Koch Institute (RKI) by the patients after completion. Primary diagnostic laboratories that participated in the study forwarded *Campylobacter* isolates of patients to Hannover Medical School (MHH) for further characterisation. If patients agreed to the analysis of their bacterial isolate in writing, multilocus sequence typing (MLST) analysis of the *Campylobacter* isolate was performed at MHH. Results were uploaded to a SeqSphere (Ridom Bioinformatics GmbH; Münster, Germany) database. Questionnaire and respective *Campylobacter* isolate could be matched using the sample number given by the primary diagnostic laboratory.

Controls were frequency-matched to cases by age group and federal state. Intended ratios were 1 case: 1 control in persons ≥15 years of age, and 1: 4 in persons younger than 15 years of age, based on sample size calculations and expected low number of cases among children. Control persons were selected in a two-step randomised procedure from address lists provided by regional population registries^26^. The self-administered questionnaires were sent out to potential control persons every month during the study period proportional to the number of expected cases, which was estimated based on surveillance data of previous years. Participating control persons returned the completed questionnaire free of charge to the RKI by mail.

### Data Collection

Cases and controls were queried about potential risk factors with a focus on consumption of certain food items. Questions on eating habits, kitchen hygiene, eating out, contact with animals, leisure activities, occupational exposure (e.g., to raw meat or young children), medication, certain chronic illnesses, travel abroad, and basic demographics (e.g., sex, month and year of birth, postal code, level of professional education, migrant background, household size) were also included. Questions about possible exposures referred to the 7 days before disease onset (case patients) or before completion of the questionnaire (controls), unless stated otherwise (see Supplementary Information: Questionnaire). Parents/caregivers were asked to complete the questionnaire for, or when appropriate, with their children.

### Data Analysis

Data was entered into an EpiData database (version 3.1, The EpiData Association, Denmark) and validated by double data entry. Missing data on sex, age, and date of disease onset of case patients was supplemented with data obtained from the national surveillance database of notified cases hosted at the Robert Koch Institute, if possible. Missing answers in item lists of the questionnaire were converted to “No” answers as described before^26^. According to the definition of the German Federal Statistical Office^27^, persons who were borne with a non-German citizenship or with at least one parent that was borne with a non-German citizenship were considered as persons with a migrant background. Seasons were categorised as follows: spring (March-May), summer (June-August), autumn (September-November), winter (December-February).

Data was analysed with Stata 14 (Stata Corporation, USA). For risk factor analyses cases were defined as patients with a laboratory diagnosed, notified *Campylobacter* enteritis. Cases were excluded if they had travelled abroad in the 7 days before the onset of illness, or if the time period between onset of illness and completion of the questionnaire was 60 days or longer. Controls were excluded from data analysis if they had travelled abroad in the 7 days before completing the questionnaire. We conducted unconditional logistic regression analyses based on single exposure variables adjusted for sex and the two matching variables age group (0–4, 5–14, ≥15 years) and federal state (“univariable analyses”) to determine adjusted odds ratios (aOR) with 95% confidence intervals (CI). Statistical significance was assessed using Wald tests. Variables were considered for multivariable analysis (MVA) if the *P*-value was 0.1 or lower in univariable analysis. To reduce the overall number of variables in the starting set for MVA, correlating variables measuring related exposures were combined to one composite variable, when plausible, or only one of the correlating variables was chosen for model building. MVA was conducted as described before^26^. Matching variables and the variable sex were forced into the model. The age group variable was modified (0–2, 3–4 years) in the multivariable model for identification of risk factors in children under 5 years of age. When building models for identifying risk factors for *Campylobacter* infections at the species level (*C. jejuni* or *C. coli*), we only included cases with isolates confirmed as either *C. jejuni* or *C. coli* by detailed molecular and biochemical analysis at MHH. The model for identifying risk factors for *C. coli* infections was limited to the age group ≥15 years because only 2 confirmed *C. coli* infections occurred in younger age groups. A modified age group variable (15–29, 30–59, 60 + years) was included in this model. We compared multivariable logistic regression models with and without exclusion of cases and controls that had travelled abroad.

We also determined risk factors for *Campylobacter* infections attributed to the source chicken by our source attribution model described below. In this approach, we conducted univariable and multivariable logistic regression analyses, comparing cases that were attributed to chicken with a relative posterior probability (*Pr*) of 0.5 or higher (n = 486; mean *Pr* 0.76, range 0.50–0.90) with controls (n = 3,983). The number of human isolates attributed to other putative sources based on *Pr* ≥0.5 was too small to allow meaningful logistic regression analysis for the identification of source-specific risk factors (pig: n = 24; mean *Pr* 0.81, range 0.55–1.00; pet: n = 19; mean *Pr* 0.68, range 0.50–0.79; poultry other than chicken: n = 4; mean *Pr* 0.65, range 0.53–0.83); cattle: n = 0).

Population attributable fractions (PAF) of each statistically significant risk factor in the final models were determined as described by Bruzzi *et al*.^28^. Confidence intervals of population attributable fractions were calculated in R, version 3.2.3^29^, based on the percentile method for samples obtained by an age-group and federal-state stratified bootstrap^30^.

### *Campylobacter* isolates from animal, food, and environmental samples

Various animal and environmental samples were obtained and food items for sampling were purchased in stores in the study region (Berlin and Brandenburg) within the study time period (total number of samples: 1,471). *Campylobacter* was isolated from a total of 183 samples. A selection of the *C. jejuni* (n = 77) and the *C. coli* isolates (n = 34) were collected at MHH and further analysed using MLST^31, 32^. Additional *C. jejuni* and *C. coli* isolates from chicken meat samples (n = 67) from the study region were provided by the National Reference Laboratory for *Campylobacter* at the Federal Institute for Risk Assessment, Berlin, Germany, and also analysed using MLST at MHH.

### Multilocus Sequence Typing (MLST)

MLST of *Campylobacter* isolates was performed using the *C. jejuni*/*C. coli* typing system developed by Dingle *et al*.^31^ and primer sequences available from http://pubmlst.org/campylobacter/info/primers.shtml. Briefly, fragments from 7 housekeeping genes, *aspA*, *glnA*, *gltA*, *glyA*, *pgmA*, *tkt*, and *uncA*, were PCR-amplified from purified genomic DNA of *C. jejuni or C. coli* isolates, and sequenced from both strands on an ABI 3130xl capillary sequencer. Sequence reads were imported into a SeqSphere+ (Ridom Bioinformatics GmbH, Münster, Germany) database for further processing and assignment to known sequence types (STs). Novel allele sequences and isolates with novel combinations of alleles were submitted to the PubMLST *Campylobacter* database (http://pubmlst.org/campylobacter/) sited at the University of Oxford^33^, to obtain allele and ST numbers. All MLST profiles for isolates newly described in this study have been deposited at the PubMLST database (www.pubmlst.org). Minimal spanning trees were generated using BioNumerics 7.1 (Applied Maths, Sint-Martens-Latem, Belgium).

### Source attribution analysis based on MLST data

MLST-typed human isolates (n = 613) were compared to 504 MLST-typed isolates from animal and food samples that were obtained in the study region during the study period or were obtained in Germany in the time period 2006–2010 in a previous study performed within the FBI-Zoo network^32^. As sources relevant for Germany we considered chicken, pig, cattle, pet (dog or cat), and poultry other than chicken (“other poultry”: duck, goose, turkey, or quail). We increased the number of animal and food isolates for our source attribution analyses by supplementing sequence type data from isolates of the same 5 sources obtained in Germany (outside the FBI-Zoo network) and neighbouring European countries (Switzerland, The Netherlands, Luxembourg, France, Belgium) in 2003 or later that were available in the PubMLST *Campylobacter* database. A large proportion of these isolates had been used for source attribution analysis in studies conducted in Luxembourg^14^ and Switzerland^11^. A total of 2,549 animal and food isolates were included (766 from Germany, 1,783 from neighbouring countries) (Table 1). Source attribution was performed using Bayesian inference on an asymmetric island model^7^ as implemented in the iSource program available from http://www.danielwilson.me.uk/iSource.html. This analysis estimates relative posterior probabilities for each human isolate to originate from the different sources. In one approach, we excluded the source pet and restricted the sources that were considered for attribution to chicken, pig, cattle, and poultry other than chicken (“other poultry”).

**Table 1:** Origin of *Campylobacter* isolates (typed with MLST) used for source attribution analysis.

Source	Germany (FBI-Zoo network: 2011–2014)	Germany (FBI-Zoo network 2006–2010^32^)	Germany (PubMLST, 2003 or later)	Luxembourg (PubMLST^14^; includes pig isolates from Belgium, France)	Switzerland (PubMLST^11^)	The Netherlands (PubMLST; 2003 or later)	Total
Human	613	—	—	—	—	—	613
Pet (Dog or Cat)	21	4	2	—	140	—	167
Chicken	90	136	138	252	540	350	1,506
Pig	47	106	9	45	257	17	481
Cattle	1	40	61	101	—	5	208
Other Poultry	29	30	52	62	—	14	187

The source “other poultry” includes isolates from ducks (n = 47), turkeys (n = 102), geese (n = 11), and quails (n = 27).

We validated our model with 32 down-sampled datasets in each of which a random subset of 10% of animal/food isolates were excluded to attribute the origin of the human isolates. Mean posterior source probability of the 5 sources was calculated. Results remained relatively stable, which means that the output did not depend much on exactly which animal/food isolates were used for analysis (Supplementary Fig. S5).

## Results

### Study population

We received 2,073 questionnaires from case patients, corresponding to 22% of all cases notified to the local health authorities in the study region, and to 68% of patients that had received a questionnaire from the local health authority. Participation of parents of children <5 years of age was slightly lower (20%) than participation of parents of older children (22%) and of persons ≥15 years of age (23%). Comparing participating campylobacteriosis cases to all non-participating cases that were notified to local health authorities in the study region, we found that they were similar in age, but a slightly higher proportion of participants was female (participants: median age 40 years; interquartile range (IQR) 24–54 years; 52% female; non-participants: median age 35 years; IQR 22–54 years; 47% female). For risk factor analyses, we excluded case patients that had travelled abroad in the 7 days before disease onset (n = 246, 12%), or had completed the questionnaire ≥60 days after onset of disease (n = 15, 0.7%), which resulted in a total of 1,812 case patients included in data analysis (Table 2). Most cases (84%) were in the age group 15 years and older, mainly because the number of notified *Campylobacter* infections was substantially lower in the younger age groups. The median time interval between disease onset and completing the questionnaire was 16 days (IQR: 12–22 days).

**Table 2:** Characteristics of the study population.

Characteristics	Case patients n (%)	Control persons n (%)
Total	1,812 (100)	3,983 (100)
Age group		
0–4 years	119 (7)	878 (22)
5–14 years	161 (9)	1,010 (26)
15 years and older	1,532 (84)	2,027 (52)
Sex		
Male	875 (48)	1,825 (47)
Female	937 (52)	2,093 (53)
Federal state		
Berlin	1,030 (57)	2,021 (51)
Brandenburg	669 (37)	1,234 (31)
Saxony	87 (5)	495 (12)
North Rhine-Westphalia	26 (1)	232 (6)

Case-control study, Germany, 2011–2014.

Of the 16,287 potential control persons that were mailed a questionnaire, 4,196 (26%) completed it. Of those, 213 questionnaires (5%) were excluded because the person had travelled abroad. The resulting control group comprised 3,983 persons. In the study population, the proportion of female and male persons was similar among cases (52% female) and controls (53% female). Controls were younger than cases because the control to case ratio was higher in the age groups <15 years (Table 2).

### Clinical aspects

Symptoms of *Campylobacter* infection in case patients were diarrhoea (95%), abdominal pain (81%), fever (53%), nausea (48%) and vomiting (19%). 25% of case patients reported bloody stools. Additional symptoms, such as headaches, chills, body aches, and weakness were reported by 62% of patients. Median duration of symptoms was 6 days (IQR 5–9 days). 18% of case patients were hospitalised because of their *Campylobacter* infection; median duration of hospital stay was 4 days (IQR 3–6 days). The proportion of case patients that reported bloody stools, fever, nausea or vomiting was higher in the hospitalised than in the non-hospitalised group. About one third of case patients (31%) reported treatment of their *Campylobacter* infection with antibiotics. The most frequently named antibiotics were ciprofloxacin (45%) and erythromycin (21%). In total, 79% of patients at working age (15–64 years) or working parents of children reported absence from work due to the illness or the illness of their child, respectively, for a median of 6 days (IQR 4–9 days).

### Risk factor analyses

Exposures related to consumption of poultry, in particular consumption of chicken meat, and to preparation of poultry in the household were positively associated with *Campylobacter* infection in univariable analyses. Other variables that were positively associated included, e.g., contact with chickens or ducks and geese; eating out; contact with sand in a sandbox; and use of gastric acid inhibitors. An exposure implying inadequate kitchen hygiene (simultaneous preparation of raw meat and food items eaten uncooked, e.g., raw vegetables, fruit, lettuce), but also exposures implying good kitchen hygiene (frequently or always using separate utensils for raw meat and other food items; frequently or always using a dishwasher for utensils that came in contact with raw meat) were positively associated with disease. A variety of variables were negatively associated with *Campylobacter* infection in univariable analyses, e.g., mostly vegetarian lifestyle; consumption of fresh fruit or herbs, raw milk, or food items purchased directly at a farm; mostly or always cleaning kitchen utensils with hot water after preparing raw meat; swimming in a pool; attending day care; medium or high professional education.

In the multivariable logistic regression model, consumption of chicken meat (aOR 1.6; population attributable fraction (PAF) 31%) and eating out (aOR 1.6; PAF 30%) were the most important risk factors for *Campylobacter* infections according to PAF, which corresponds to the proportion of cases that could be avoided in the population if this risk factor was eliminated (Table 3). Preparation of packaged poultry meat in the household (PAF 14%), simultaneous preparation of raw meat and food items consumed uncooked (PAF 12%), and contact with poultry animals (PAF 3%) were risk factors as well (Table 3). The use of gastric acid inhibitors in the past 4 weeks was also positively associated with *Campylobacter* infection (aOR 1.9; PAF 10%). We found a negative association with disease in the final model for a mostly vegetarian lifestyle (aOR 0.5); consumption of beef (aOR 0.7); consumption of lamb/mutton (aOR 0.6); consumption of fruit (aOR 0.6); contact with a dog (aOR 0.8); and recreational swimming (aOR 0.7) (Table 3). Results of multivariable analysis were similar when the model was restricted to *C. jejuni* infections, except that the association of contact with poultry animals and disease was no longer statistically significant (data not shown).

**Table 3:** Factors positively associated (risk factors) and factors negatively associated with *Campylobacter* infections.

Exposure	Cases Exposed % (n)	Controls Exposed % (n)	aOR^a^ (95% CI^b^)	Population Attributable Fraction % (95% CI^b^)
Consumed any chicken meat***	87.0 (1,445/1,661)	79.1 (2,967/3,753)	1.6 (1.2–2.0)	31 (17–42)
Ate out (at food stand, restaurant, canteen, etc.)***	81.9 (1,437/1,755)	78.6 (3,089/3,929)	1.6 (1.3–2.0)	30 (18–40)
Prepared poultry meat (packaged) in household***	53.9 (860/1,597)	43.8 (1,617/3,692)	1.4 (1.1–1.6)	14 (8–20)
Prepared uncooked food and raw meat in household at the same time**	52.0 (856/1,646)	45.8 (1,684/3,677)	1.3 (1.1–1.5)	12 (4–18)
Used anti-acidic drug (PPI)***	21.1 (371/1,755)	8.1 (315/3,869)	1.9 (1.5–2.3)	10 (7–12)
Had contact with poultry (animal)***	5.3 (92/1,725)	4.4 (170/3,856)	2.1 (1.4–3.0)	3 (2–4)
Consumed mostly vegetarian food*	1.5 (25/1,646)	4.1 (151/3,669)	0.5 (0.3–1.0)	—
Consumed (unpeeled) fruit***	62.8 (1,055/1,679)	72.7 (2,757/3,794)	0.6 (0.5–0.7)	—
Consumed lamb/mutton**	8.0 (129/1,615)	8.5 (321/3,767)	0.6 (0.5–0.9)	—
Consumed beef***	51.1 (793/1,551)	52.6 (1,923/3,654)	0.7 (0.6–0.8)	—
Had contact with dog**	29.0 (498/1,716)	32.8 (1,256/3,828)	0.7 (0.6–0.9)	—
Went swimming (in pool, lake, ocean, etc.)**	14.6 (257/1,755)	23.4 (913/3,910)	0.7 (0.6–0.9)	—

Case-control study, Germany, 2011–2014. The proportion of exposed cases and controls was based on the number of cases and controls with complete answers in univariable analysis (without adjustment for age group, sex, federal state). Adjusted odds ratios (aOR) were determined in multivariable logistic regression analysis (adjusted for age group, sex, federal state; 1,003 cases and 2,569 controls with complete answers for all variables in the final model). ^a^Adjusted odds ratio. ^b^Confidence interval. *Indicates *P* < 0.05. **Indicates *P* < 0.01. ***Indicates *P* < 0.001.

In an analysis restricted to children under 5 years of age, contact with sand in a sandbox (PAF 39%), preparation of poultry meat (fresh or packaged) in the household (PAF 38%), contact with poultry animals (PAF 22%), and a migrant background (PAF 10%) were positively associated with a *Campylobacter* infection. Consumption of chicken meat was not a statistically significant risk factor for this age group. No exposure variables were negatively associated with disease in this analysis (Table 4).

**Table 4:** Risk factors for *Campylobacter* infections in children <5 years of age.

Exposure	Cases Exposed % (n)	Controls Exposed % (n)	aOR^a^ (95% CI^b^)	Population Attributable Fraction % (95% CI^b^)
Had contact with sand (in a sandbox or similar)^c^	85.5 (94/110)	76.5 (643/841)	1.9 (1.0–3.5)	39 (0–63)
Prepared poultry meat (fresh or packaged) in household*	78.0 (85/109)	61.8 (525/850)	2.0 (1.2–3.4)	38 (11–56)
Had contact with poultry (animal)***	24.1 (26/108)	6.2 (52/835)	5.2 (2.9–9.5)	22 (17–24)
Migrant background**	13.7 (16/117)	11.6 (100/862)	2.7 (1.4–5.5)	10 (4–13)

Case-control study, Germany, 2011–2014. The proportion of exposed cases and controls is based on the number of cases and controls with complete answers in univariable analysis (without adjustment for age group, sex, federal state). Adjusted odds ratios (aOR) were determined in multivariable logistic regression analysis (adjusted for age group (0–2 years, 3–4 years), sex; federal state; 90 cases and 762 controls with complete answers for all variables in the final model). No factors were negatively associated with disease in the final model. ^a^Adjusted odds ratio. ^b^Confidence interval. ^c^ *P* = 0.054. *Indicates *P* < 0.05. **Indicates *P* < 0.01. ***Indicates *P* < 0.001.

In an analysis to identify risk factors for *C. coli* infections, consumption of pork (PAF 66%) and the use of gastric acid inhibitors in the past 4 weeks (PAF 20%) were positively associated with disease, whereas consumption of beef and consumption of fruit were negatively associated (Table 5).

**Table 5:** Factors positively associated (risk factors) and factors negatively associated with *Campylobacter coli* infections in age group ≥15 years.

Exposure	Cases Exposed % (n)	Controls Exposed % (n)	aOR^a^ (95% CI^b^)	Population Attributable Fraction % (95% CI^b^)
Consumed pork*	95.1 (58/61)	83.8 (1,690/2,016)	3.3 (1.0–11.0)	66 (18–94)
Used anti-acidic drug (PPI)**	31.8 (20/63)	15.1 (305/2,023)	3.1 (1.6–5.9)	20 (11–25)
Consumed (unpeeled) fruit**	54.2 (32/59)	71.7 (1,461/2,037)	0.4 (0.2–0.8)	—
Consumed beef*	46.3 (25/54)	56.2 (1,106/1,967)	0.5 (0.3–0.9)	—

Case-control study, Germany, 2011–2014. The proportion of exposed cases and controls is based on the number of cases and controls with complete answers in univariable analysis (without adjustment for age group, sex, federal state). Adjusted odds ratios (aOR) were determined in multivariable logistic regression analysis (adjusted for age group (15–29 years, 30–59 years, ≥60 years), federal state, sex; 50 cases and 1,786 controls with complete answers for all variables in the final model). ^a^Adjusted odds ratio. ^b^Confidence interval. *Indicates *P* < 0.05. **Indicates *P* < 0.01.

When we performed the same multivariable analysis including cases and controls that had travelled abroad and with an additional exposure variable “travelled abroad”, the results did not change substantially. Travelling abroad was positively associated with *Campylobacter* infection in the model for all age groups (*P* < 0.001; Supplementary Table S1), and in the models for *C. jejuni* (*P* < 0.001) and *C. coli* infections (*P* < 0.05). Travelling abroad was not statistically significantly associated with *Campylobacter* infection in the model for children <5 years of age (data not shown).

We also performed multivariable logistic regression analysis with cases whose *Campylobacter* isolate had been attributed to the source chicken with a probability of 50% or higher in the source attribution model (n = 486) and the controls (n = 3,983). Risk factors and strengths of associations were similar to the model with all 1,812 cases: consumption of chicken meat (aOR 1.9); eating out (aOR 1.4); preparation of packaged poultry meat in the household (aOR 1.6); simultaneous preparation of raw meat and food items consumed uncooked (aOR 1.4); use of gastric acid inhibitors (aOR 1.8). In the final model, contact with poultry animals and having gone swimming were not statistically significantly associated with *Campylobacter* infections attributed to chicken (Supplementary Table S2). Compared to the model with all cases (Table 3), population attributable fractions calculated for consumption of chicken meat (41% vs. 30%), preparation of poultry meat in the household (21% vs. 14%) and simultaneously preparing uncooked food and raw meat in the household (17% vs. 12%) were higher, whereas the PAF for eating out was lower (25% vs. 30%), and the PAF for use of gastric acid inhibitors was about the same (11% vs. 10%) (Supplementary Table S2).

Due to the small number of human isolates that were attributed to sources other than chicken considered in our source attribution model (pigs, pets, cattle, poultry other than chicken), multivariable analyses similar to the ones we performed for chicken could not be conducted. In univariable analyses (not adjusted for age group, sex, federal state) we found statistically significant associations of *Campylobacter* infections attributed to pig (n = 24) and consumption of pork (100% of cases (with complete answers) had consumed pork (23/23; OR could not be calculated) vs. 85% of controls (3,220/3,787); *P* < 0.001), but not a statistically significant association with consumption of chicken (OR 0.95; *P* = 1.0). The majority of case patients with isolates assigned to the source pig reported both consumption of pork and chicken (77%; 17/22). None of the case patients with *Campylobacter* isolates assigned to the source pet (dog or cat; n = 10) reported contact with a dog (versus 33% of controls) and 3/10 reported contact with a cat (versus 33% of controls; OR 0.9; *P* = 1.0).

### Multilocus Sequence Typing

**Figure 1:**
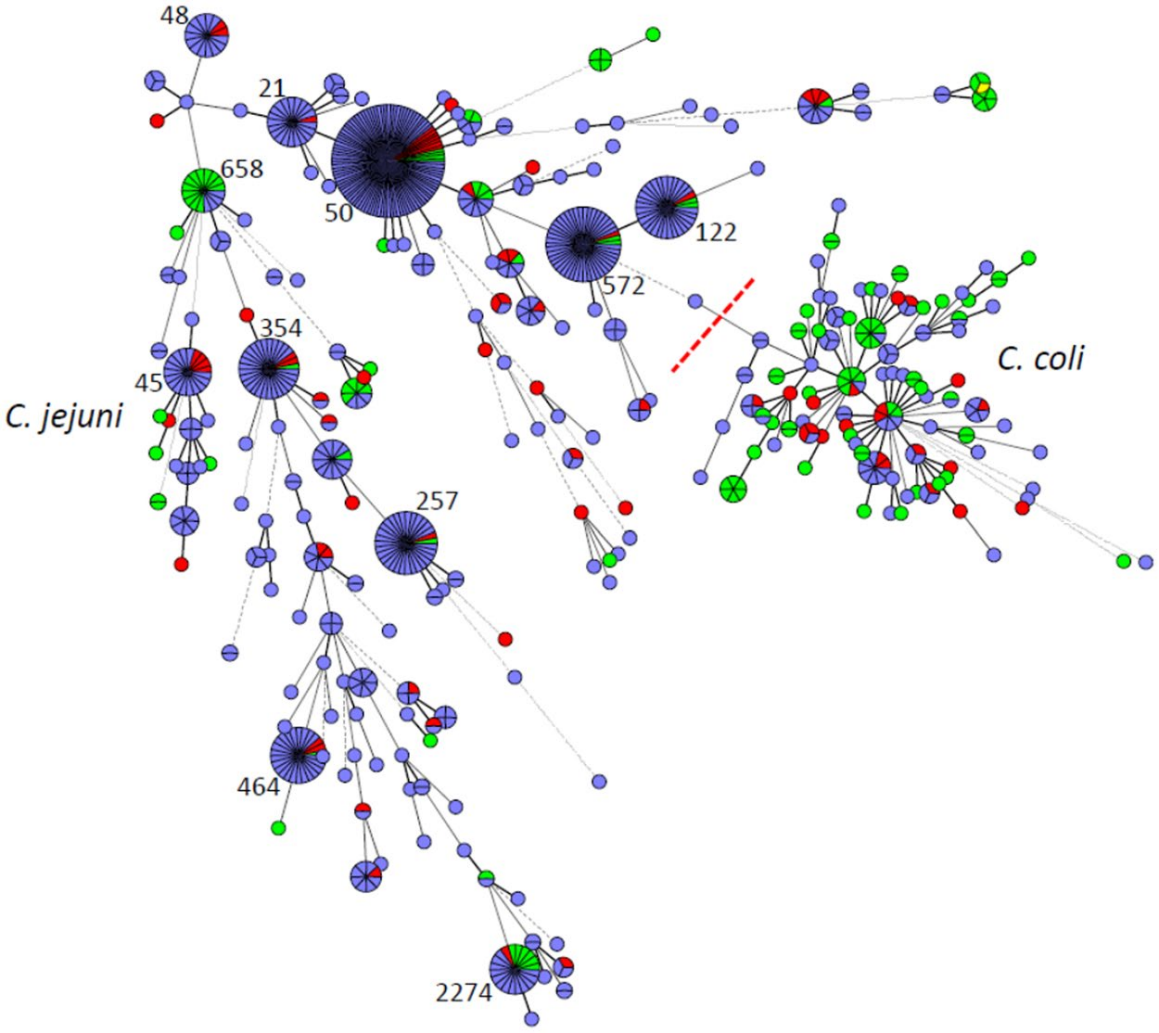
Minimal Spanning Tree generated from MLST comparisons of 816 *C. jejuni* and *C. coli* isolates from human study participants and from animals and food samples from the study region. Colouring according to isolate source: blue, isolates from patients; green, isolates from animals; red, isolates from food samples. Only abundant sequence types (STs) are labelled. A version of the figure with full labelling is available as Supplementary Figure S1.

**Figure 2:**
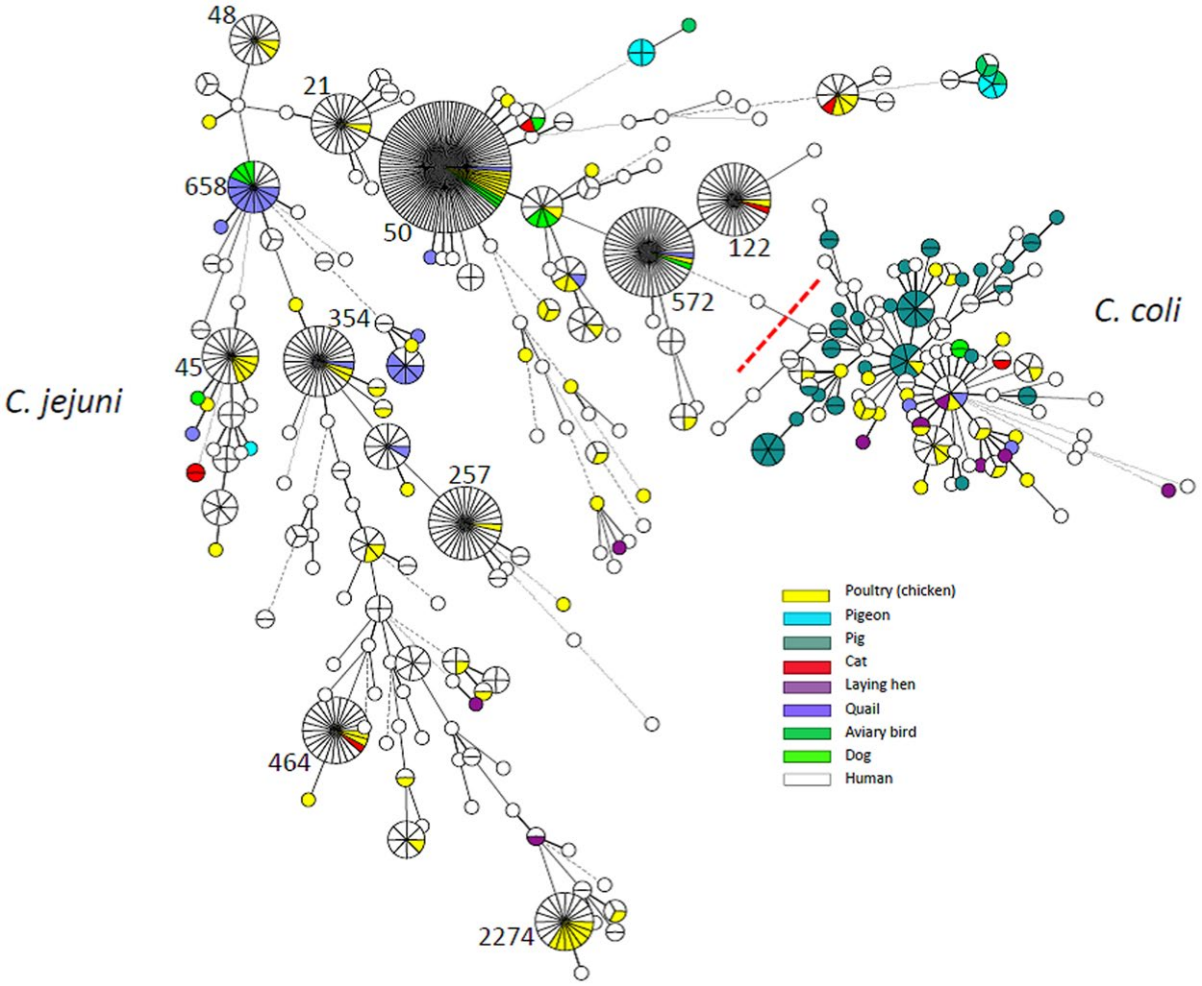
Minimal Spanning Tree generated from MLST comparisons of 816 *C. jejuni* and *C. coli* isolates from human study participants and from animals and food samples from the study region. Colouring according to host species as specified in colour legend. Only abundant sequence types (STs) are labelled. A version of the figure with full labelling is available as Supplementary Figure S2.

In total, 613 human *Campylobacter* isolates that could be matched to a questionnaire completed by the respective case patient and an additional 203 isolates from animal and food samples were characterised by MLST. The human isolates were assigned to 186 different sequence types (STs); 28 of those STs were more prevalent (ranging from 4 to 91 isolates each), whereas 158 STs were detected only 1–3 times (34% of isolates). 550 of the 613 isolates could be assigned to 28 different clonal complexes (CCs). *Campylobacter* sequence types (STs) that occurred most frequently among human isolates were ST-50 (15%), ST-572 (7%), ST-122 (5%), ST-257 (5%), ST-354 (4%), and ST-464 (4%). The most frequent clonal complexes were CC-21 (25%), CC-206 (13%), and CC-828 (10%). Notably, some STs occurred at high frequency in the dataset that had not been part of the 10 most common STs in the previous FBI-Zoo MLST study^32^. Examples were ST-122, which occurred in 29 samples, ST-354 (27 samples) and ST-464 (23 samples). The most prevalent STs of human isolates were detected in animal/food samples as well (Figs 1 and 2). The number of isolates from patients with a history of travel outside of Germany within 7 days before disease onset was too low for detailed analysis, but it was apparent that all abundant STs were detected both in patients with and without travel history (Supplementary Figs S3 and S4).

### Molecular source attribution on the basis of MLST

**Figure 3:**
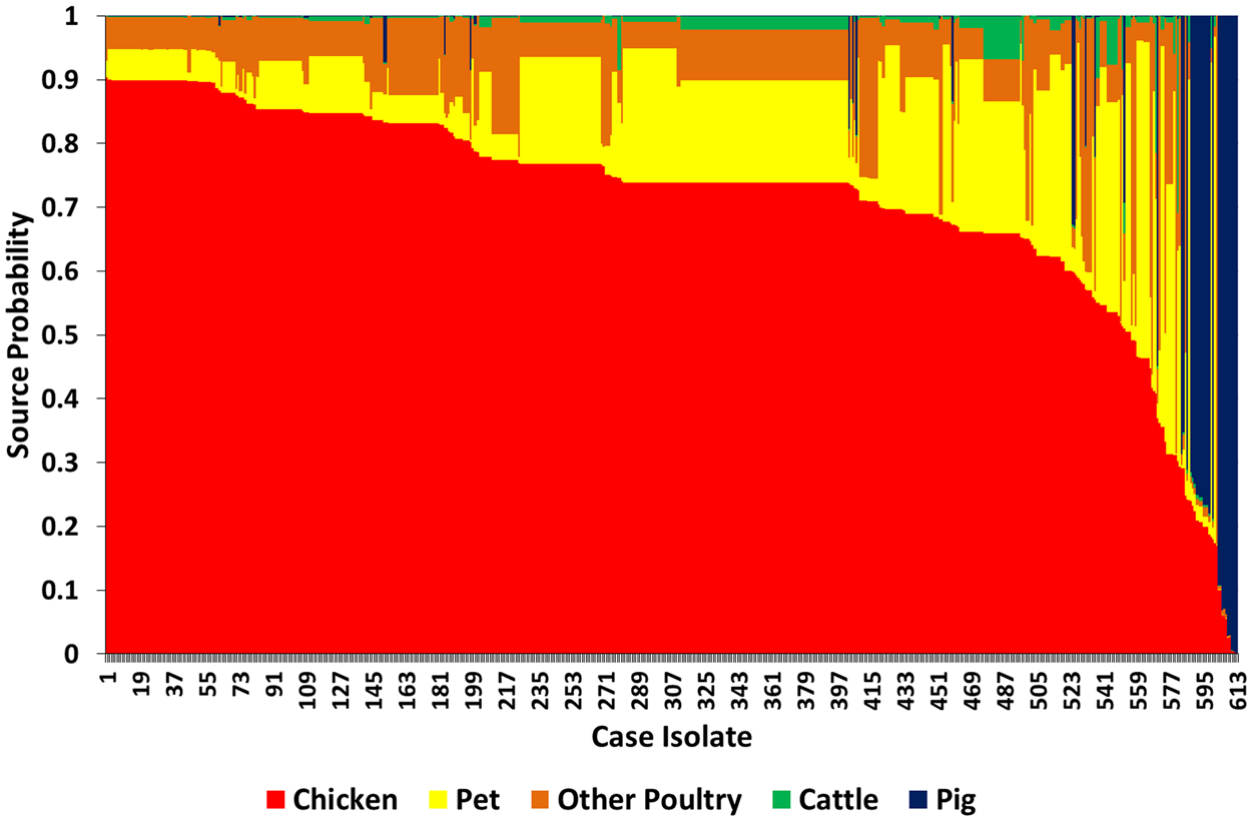
Source probabilities for human isolates (n = 613) to originate from each of the five sources (chicken, pet, pig, cattle, poultry other than chicken) as determined by source attribution analysis. MLST data from animal and food isolates obtained in Germany and in neighbouring European countries was used for source attribution analysis (Table 1).

The MLST typing data of human isolates from the case patients of the case-control study and time-matched animal and food isolates was used to perform source attribution of each human isolate. This analysis was based on an asymmetric island model for which Bayesian inference is implemented in the software iSource^7^. The output is a matrix of posterior probabilities of each human isolate originating from each of the putative sources (Fig. 3). We considered 5 putative sources: chicken, pig, pet dog or cat, cattle, other poultry (turkey, duck, goose, quail). To ensure that the population of each source was well characterised, we supplemented the German MLST data with publicly available MLST data obtained in neighbouring European countries. When using a 50% cut-off on the posterior probability of origin, 91% (555/613) of human isolates were attributed to chicken, 4% (27/613) to pig, 1% (6/613) to pet, and 0.8% (5/613) to other poultry. None of the isolates were attributed to cattle. The proportion of isolates that could not be attributed to any of the 5 sources was 3% (20/613). The mean probability of human isolates to originate from chicken was 71%, from pig 4%, from pet 14%, from cattle 1%, and from other poultry 9% (Table 6). The probability to originate from chicken was high for the most frequent STs of human isolates: 74% for ST-50, 77% for ST-572, 74% for ST-122, 85% for ST-257, 83% for ST-354, and 85% for ST-464. For *C. coli* isolates, the mean probability to originate from pig was higher, whereas the probability to originate from chicken was lower relative to *C. jejuni* isolates (Table 6). The probability to originate from pigs was almost zero for *C. jejuni* isolates (Table 6). We stratified human isolates according to sex, age group, region of living (urban or rural) of case patients, and by season of disease onset in case patients but did not find substantial differences between the strata (Table 6). When we excluded the source pet (dog or cat) from our analysis, the mean posterior probability of the human isolates to originate from the remaining 4 sources was as follows: chicken 0.82; pig 0.04; cattle 0.03; poultry other than chicken 0.11.

**Table 6:** Mean posterior probabilities of human isolates of originating from one of the putative sources (chickens, pigs, pet dogs or cats, cattle, poultry other than chicken (duck, goose, turkey, quail: “other poultry”)) as determined by asymmetric island source attribution modelling based on MLST data.

Human isolates	Mean posterior source probability				
	Chicken	Pig	Pet	Cattle	Other poultry
All human isolates (n = 613)	0.71	0.04	0.14	0.01	0.09
*Campylobacter* species					
*C. jejuni* (n = 537)	0.74	0.001	0.16	0.01	0.09
*C. coli* (n = 76)	0.56	0.32	0.04	0.004	0.08
Sex of case patient					
Female (n = 334)	0.72	0.04	0.14	0.01	0.09
Male (n = 279)	0.71	0.05	0.14	0.01	0.09
Age group of case patient					
0–4 years (n = 17)	0.70	<0.001	0.16	0.02	0.12
5–14 years (n = 32)	0.71	0.02	0.15	0.01	0.09
≥15years (n = 564)	0.72	0.04	0.14	0.01	0.09
Region of living of case patient					
Urban (n = 445)	0.72	0.03	0.14	0.01	0.10
Rural (n = 168)	0.69	0.08	0.14	0.01	0.08
Season of disease onset in case patient					
Spring (n = 123)	0.71	0.05	0.13	0.01	0.08
Summer (n = 247)	0.73	0.03	0.14	0.01	0.09
Autumn (n = 108)	0.70	0.06	0.13	0.01	0.09
Winter (n = 135)	0.69	0.04	0.15	0.01	0.10

Stratification according to *Campylobacter* species or characteristics of the case patients. Due to rounding of numbers the sum of probabilities may not add up to 1.00.

## Discussion

We describe the first combined case-control and source attribution study for *Campylobacter* infections in Germany. We identified consumption of chicken meat, eating out, preparation of packaged chicken meat in the household and contact with poultry animals, as significant risk factors for *Campylobacter* infections. The use of gastric acid inhibitors was also positively associated with *Campylobacter* infection. Consumption of chicken meat was the most important risk factor for *Campylobacter* infections with a population attributable fraction (PAF) of 31%, confirming results from studies conducted in other countries^10, 14, 18–21, 23^. Chicken meat is frequently contaminated with *Campylobacter*. 25% of caecum samples and 52% of neck skin samples taken from broilers at abattoirs tested positive for *Campylobacter* in a zoonosis monitoring by veterinary authorities in Germany^34^. The higher prevalence on skin samples indicated that the carcasses were contaminated during slaughtering. A high proportion of samples (38–54%) taken from fresh broiler meat at retail also tested positive for *Campylobacter* ^2, 34, 35^.

Eating out, especially eating chicken at a restaurant, has been described as a risk factor in other studies^14, 21, 22, 36–38^. Exposure to *Campylobacter* while eating out may have occurred through consumption of insufficiently heated meat, e.g., from chicken, or through cross-contamination of food items in the restaurant kitchen. Cross-contamination due to inadequate kitchen hygiene in the private household may also underlie the positive association of *Campylobacter* infection and preparation of packaged chicken meat in this study. Use of gastric acid inhibitors, such as omeprazole and pantoprazole, for other therapeutically indicated reasons not related to *Campylobacter* infection was identified as another risk factor for *Campylobacter* infections, which confirms results from previous studies on *Campylobacter* and other bacterial gastrointestinal infections^20, 21, 39–44^. The association appears plausible, because an increase in the stomach pH may result in the survival of higher bacterial loads of incoming intestinal pathogens, such as *Campylobacter*, in the stomach^45^. Patients using gastric acid inhibitors should be informed by their physicians or pharmacists about the association with *Campylobacter* or other bacterial gastrointestinal infections, so that the patients can make an informed choice to avoid eating risk food items while on this medication.

In our analysis of young children we found an association of contact with sand in a sandbox and *Campylobacter* infection, which has been demonstrated as a risk factor for infection with other gastrointestinal pathogens as well^26, 42, 46^. It remains to be elucidated whether sandboxes are the actual source of infection with these pathogens, e.g., because of contamination of the sand with animal faeces (e.g., dogs, wild birds^47^), or if infectious gastrointestinal pathogens can survive well in sandboxes and can be readily transferred from child to child, or if playing in a sandbox is a proxy for a still unidentified environmental exposure. Interestingly, consumption of chicken meat was not a statistically significant risk factor for *Campylobacter* infections in young children^48, 49^, but preparation of chicken meat (fresh or packaged) in the household was, again indicating that infections may have occurred via cross-contamination of other food items. A migrant background was associated with *Campylobacter* infections among young children. Cases and controls in this age group that reported a migrant background came from a wide variety of European and non-European countries and it is unclear why the odds of cases having a migrant background would be higher than for controls. One explanation may be that parents of healthy children with a migrant background more frequently decided against participating in our study, compared to parents of children with a migrant background that had been ill.

Besides the use of gastric acid inhibitors we identified consumption of pork as a risk factor for *C. coli* infections in persons 15 years and older. In line with this result, the mean probability of human *C. coli* isolates to originate from pig in our source attribution model was relatively high (32%). Consumption or preparation of chicken was not a risk factor for *C. coli* infections, which was unexpected because 28% of chicken meat samples taken at retail as part of the zoonosis monitoring program in Germany tested positive for *C. coli* in 2014^50^. One possible explanation is the small number of confirmed *C. coli* cases in our dataset (age group >= 15 years: n = 65), which resulted in insufficient analytical power for detection of a presumably weak association between consumption of chicken and *C. coli* infection. Interestingly, the mean probability of *C. coli* isolates to originate from chicken was higher than the mean probability to originate from pigs (56% vs. 32%). The mean probability of the *C. jejuni* isolates in our study to originate from pigs was close to zero and consumption of pork was not identified as a risk factor for *C. jejuni* infections. This finding is in accordance with results from the zoonosis monitoring program in Germany, where *C. jejuni* is rarely isolated from pig matrices^51^.

According to our source attribution model, about 90% of human isolates were attributable to chicken, albeit using a 50% cut-off. This is in line with other studies, where about 50–90% of human infections could be attributed to the chicken reservoir^7, 8, 10, 11, 13, 14, 24, 52^. Contrary to results from other studies^7, 10–14, 24, 25, 53^, none of the human isolates in our study was attributed to cattle. Campylobacteriosis outbreaks are frequently caused by consumption of unpasteurised (“raw”) milk, especially among children, implying cattle as the source for human *Campylobacter* infections in these outbreaks^54–56^. However, consumption of raw milk and other raw milk products was not a risk factor in our study or other studies on sporadic *Campylobacter* infections^22^. In univariable analyses, consumption of raw milk or raw milk products was even negatively associated with disease.

It was puzzling that some variables indicating good kitchen hygiene at home were identified as risk factors for disease in univariable analyses (frequently or always using separate utensils for raw meat and other food items; frequently or always using a dishwasher for utensils that came in contact with raw meat). Unexpected associations related to kitchen hygiene have been observed in a recent salmonellosis case-control study as well, and one interpretation was that case patients may overemphasise their hygienic behaviour in hindsight or may give answers that they think are socially desirable^39^.

Like any case-control study, ours is not without limitations. The proportion of female persons among case participants was slightly higher than among non-participants, therefore, participants may not be representative of all notified campylobacteriosis cases. Eating habits of women likely differ from those of men, which may bias the results of our study regarding the association with consumption of certain food items. However, the proportion of female persons was also higher in the control group, thus minimising this potential bias. In all case-control studies, including ours, recall bias may be an inherent problem. Cases may not have remembered consumption of particular food items as well as controls because the time period they were queried about was farther in the past than that of controls. The median time period between disease onset and completing the questionnaire was 16 days. Differential recall may result in an underestimation of the strength of the association. ﻿We supplemented our German MLST data with animal and food isolates from neighbouring countries to obtain a sufficiently large number of isolates for our source attribution model. This may have introduced a bias, because animal and food isolates from other countries and from other years may differ from those obtained in Germany in recent years^57^, especially if they are not a representative sample. We tried to minimise this bias by supplementing our MLST data with animal and food isolates only from neighbouring countries. Many of these isolates had been used in recent source attribution studies (Switzerland and Luxembourg). It is plausible to assume that consumption pattern and exposures pathways would be comparable between Germany and the directly neighbouring countries^57^.

Our source attribution analysis comes with certain limitations. We included pet animals (dogs and cats) as one of the possible sources of human isolates. However, since pets and their owners share the environment and possibly some of the food, it is possible that both human and pet *Campylobacter* isolates originate from another common reservoir, e.g., chicken, cattle, or pig. Pets could be viewed as a separate population in source attribution modelling because the asymmetric island model takes migration between source populations into account. The mean posterior probability of human isolates to originate from pets was rather small in our study (0.14), and, therefore, pets appeared not to be a relevant source. Only 10 human isolates (of 533) could be attributed to pets (using a 50% cut-off) and only 3 of the corresponding case-patients reported contact with a cat. When we excluded pets as a putative source from our attribution analysis, the mean posterior probability for human isolates to originate from chicken increased from 0.71 to 0.82, mean posterior probabilities to originate from other sources (cattle; poultry other than chicken) increased only slightly, or remained the same (pig). We did not consider sheep as a possible source of human isolates in our model because in Germany, in contrast to some other countries, e.g., New Zealand or Scotland, contact of people with sheep is limited and consumption of lamb/mutton does not play a major role as was confirmed by results from our case-control study (only about 9% of control persons had consumed lamb/mutton; about 2% of control persons had contact with sheep). Sheep were also a minor source of human infections in a study from the Netherlands^10^. Any source attribution modelling is limited by the putative animal or environmental sources that are included in the modelling, and, therefore, there is always the possibility to overlook sources of human infections.

This study was the first to investigate risk factors for sporadic *Campylobacter* infections in Germany and combine the results with source attribution analysis. We confirm that consumption and handling of chicken meat are important risk factors for campylobacteriosis. Tens of thousands of *Campylobacter* infections are notified in Germany every year. The true number of *Campylobacter* enteritis cases in the population is estimated to be about five to ten times higher^58–60^, and the burden of disease is considered as substantial^61, 62^. Therefore, efforts should be strengthened to minimise contamination of chicken meat with *Campylobacter* and to educate consumers about health risks associated with preparation and consumption of chicken meat. Examples from other countries have shown that improvements are possible if multidisciplinary approaches are undertaken^63, 64^.

## Conclusions

To reduce the risk of *Campylobacter* infections among consumers, control measures and intervention strategies should be adopted to reduce prevalence of *Campylobacter* in poultry and poultry meat along the food chain^65^. Until such measures are initiated and effective, consumers and food handlers should be better educated about risks associated with consumption and preparation of poultry and personal protection measures, such as sufficient heating of poultry meat before consumption and kitchen hygiene when handling raw poultry or other types of raw meat to avoid cross-contamination.

## Electronic supplementary material

Supplementary Information
