# Supplementary material for: A combined case-control and molecular source attribution study of human Campylobacter infections in Germany, 2011–2014
Source: Sci Rep. 2017 Jul 11;7:5139. doi: 10.1038/s41598-017-05227-x (PMC5505968; doi:10.1038/s41598-017-05227-x)
Supplement: Supplementary file 1 — Supplementary Information [file 41598_2017_5227_MOESM1_ESM.pdf]

SUPPLEMENTARY INFORMATION

A combined case-control and molecular source attribution study of human *Campylobacter* infections in Germany, 2011-2014

Bettina M Rosner, Anika Schielke, Xavier Didelot, Friederike Kops, Janina Breidenbach, Niklas Willrich, Greta Gölz, Thomas Alter, Kerstin Stingl, Christine Josenhans , Sebastian Suerbaum, and Klaus Stark

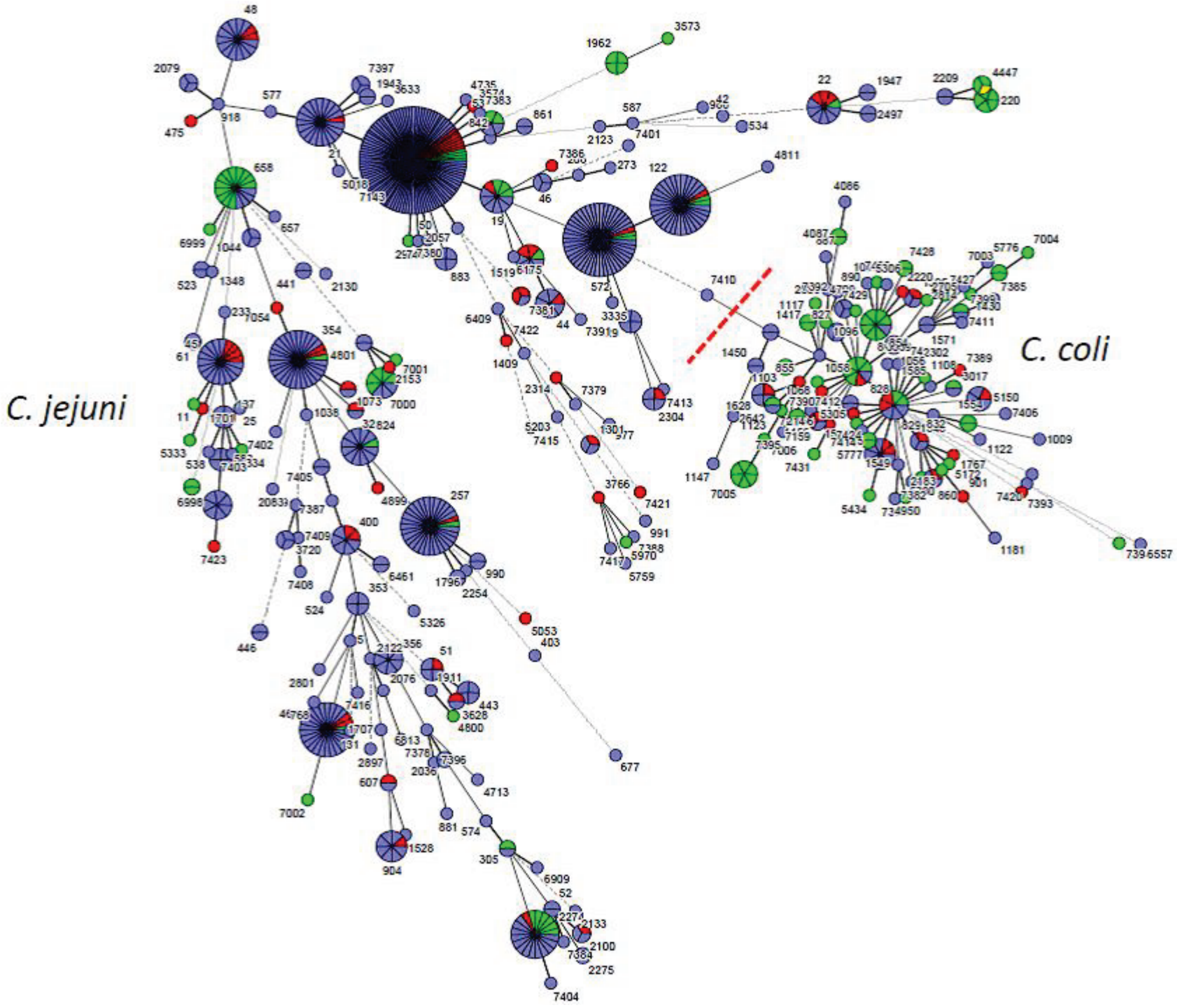

Supplementary Figure S1: Minimum Spanning Tree generated from MLST comparisons of 816 *C. jejuni* and *C. coli* isolates from human study participants and from animals and food samples from the study region. Colouring according to strain source: blue, isolates from patients; green, isolates from animals; red, isolates from food samples. Numbers close to nodes indicate sequence types (STs). See Fig. 1 for a version with only the most abundant STs labelled.

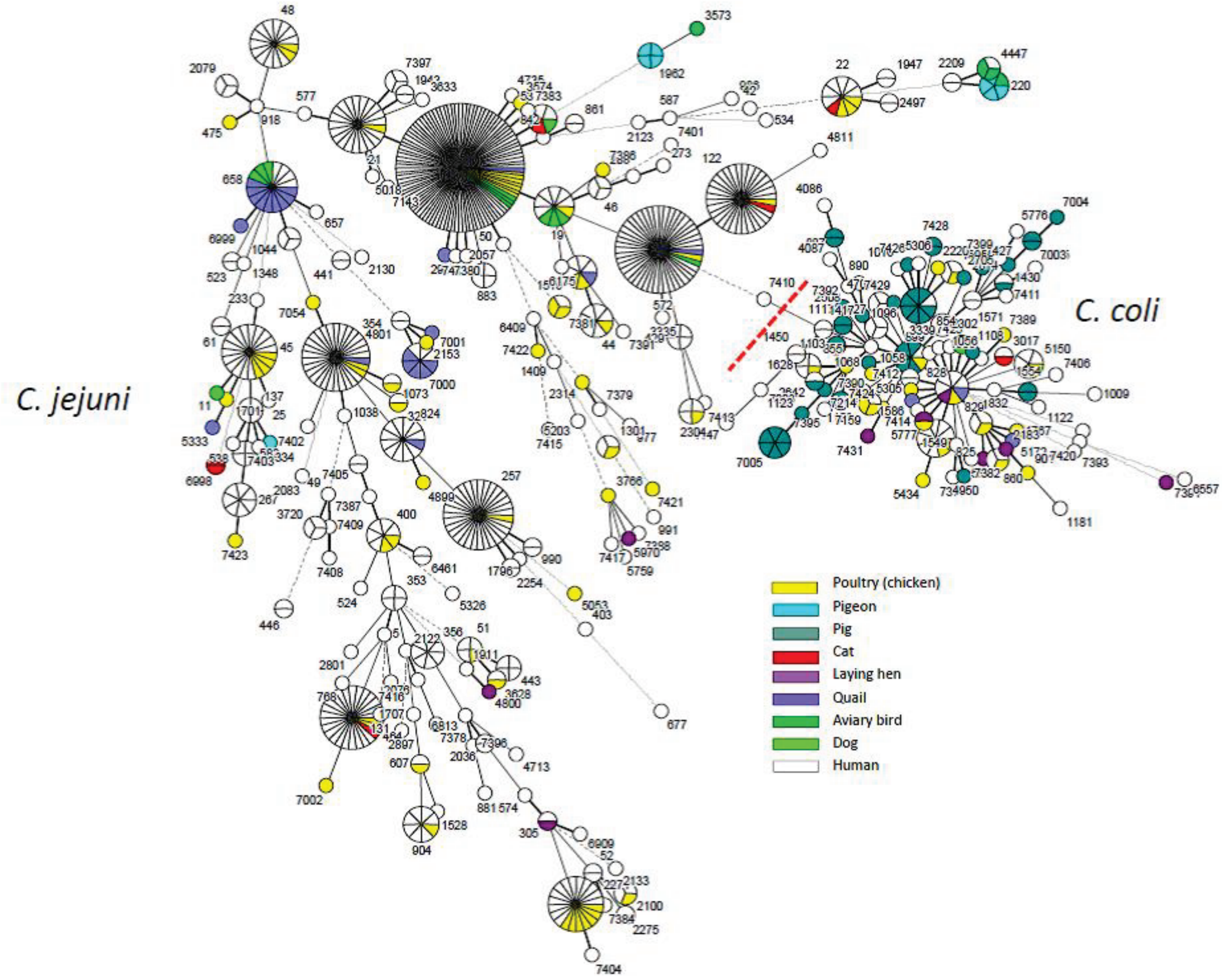

Supplementary Figure S2: Minimum Spanning Tree generated from MLST comparisons of 816 *C. jejuni* and *C. coli* isolates from human study participants and from animals and food samples from the study region. Colouring according to host species as specified in colour legend. Numbers close to nodes indicate sequence types (STs). See Fig. 2 for a version with only the most abundant STs labelled.

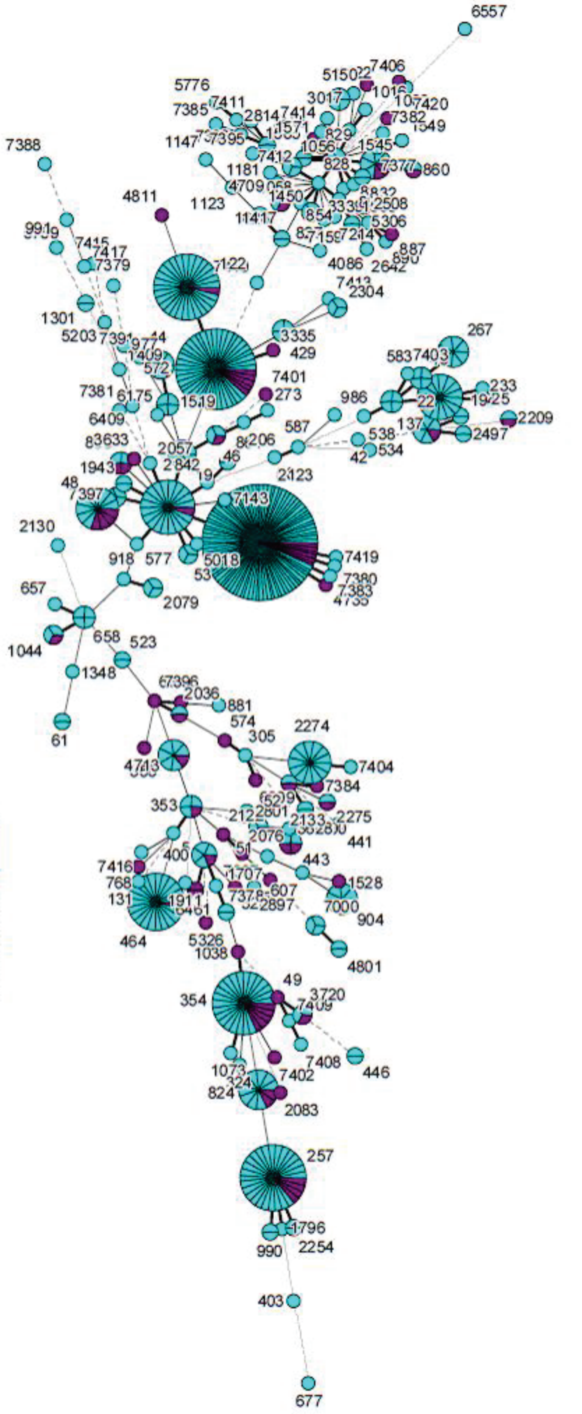

Supplementary Figure S3: Minimum Spanning Tree generated from MLST comparisons of 613 *C. jejuni* and *C. coli* isolates from human patients. Numbers close to nodes indicate sequence types. Colour coding according to travel history (outside of Germany) within 7 days prior to onset of symptoms: blue, no travel history; purple, recent travel.

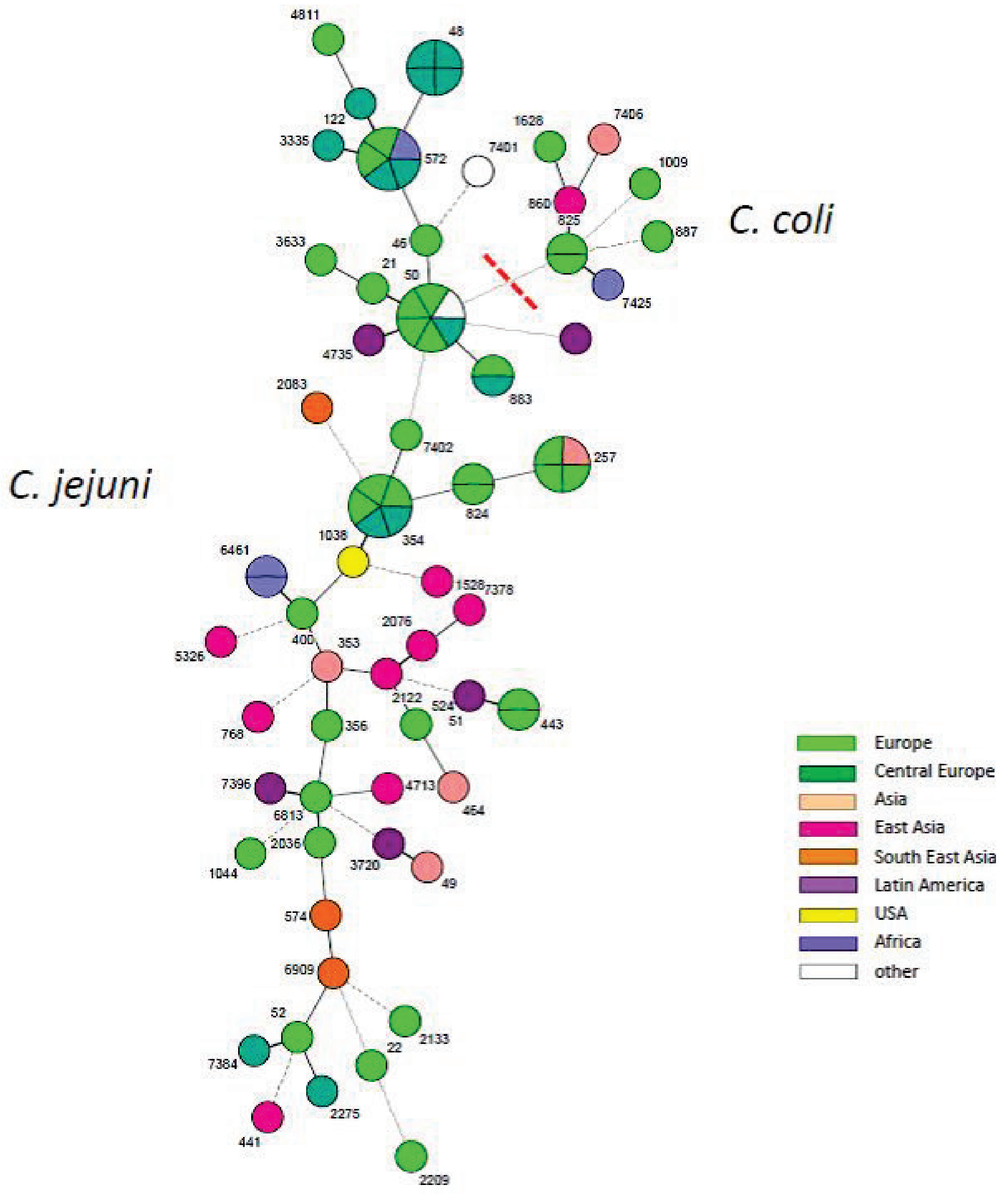

Supplementary Figure S4: Minimum Spanning Tree generated from MLST comparisons of 80 *C. jejuni* and *C. coli* isolates from human patients who had travelled outside of Germany within 7 days prior to onset of symptoms. Numbers close to nodes indicate sequence types. Colour coding indicates destination of travel, see colour legend.

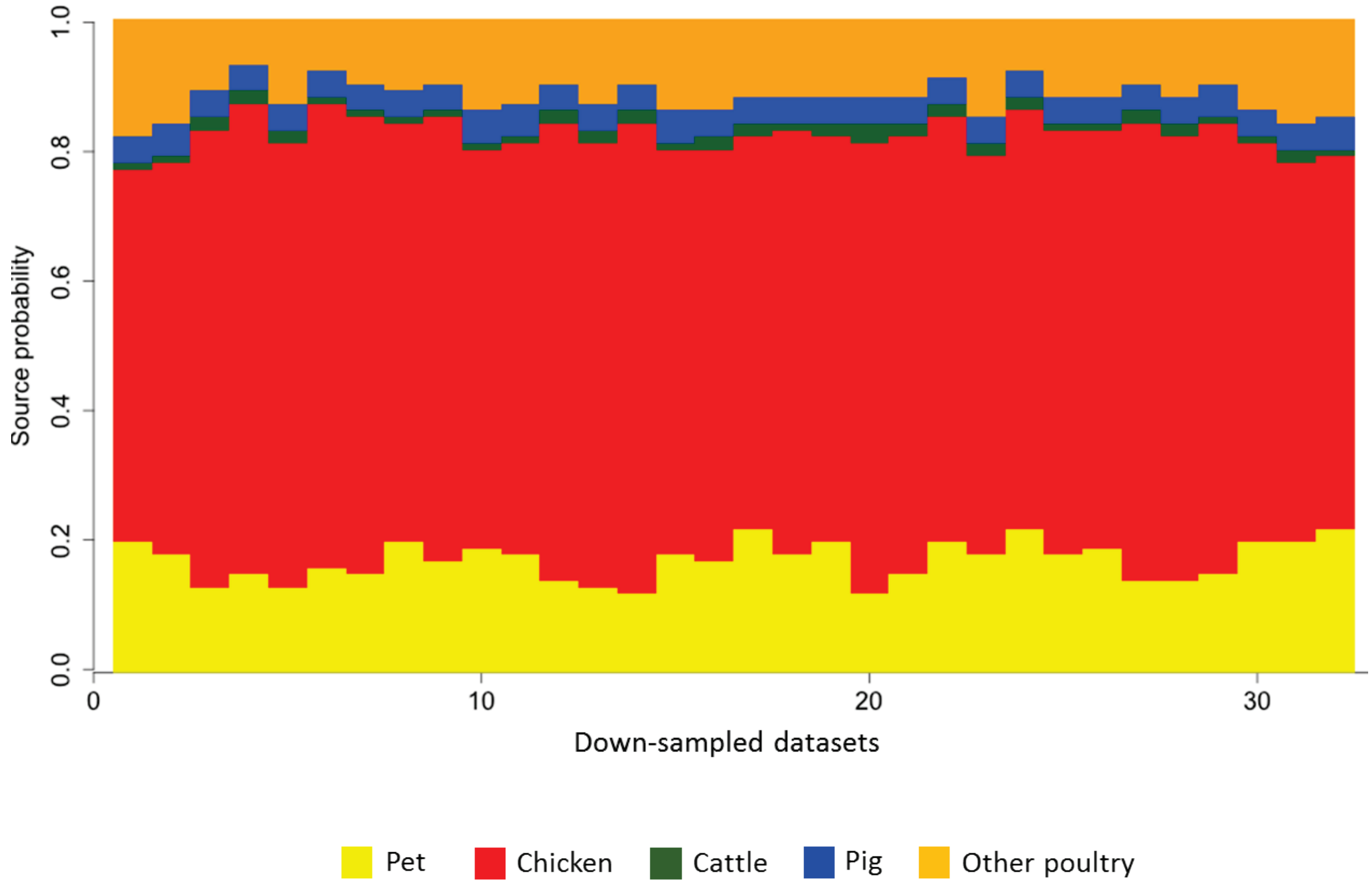

Supplementary Figure S5. Mean posterior source probability for human isolates (n=613) to originate from one of the 5 putative sources as determined by source attribution analysis with 32 down-sampled datasets. In each of the down-sampled datasets a random subsample of 10% of animal/food isolates was excluded to attribute the origin of the human isolates.

Supplementary Table S1. Factors positively associated (risk factors) and factors negatively associated with *Campylobacter* infections (international travellers included in analysis). Case-control study, Germany, 2011-2014. The proportion of exposed cases and controls is based on the number of cases and controls with complete answers in univariable analysis (without adjustment for age group, sex, federal state). Adjusted odds ratios (aOR) were determined in multivariable logistic regression analysis (adjusted for age group, sex, federal state; 1,110 cases and 2,719 controls with complete answers for all variables in final model).

Risk Factor	Cases exposed % (n)	Controls exposed % (n)	aOR[a] (95% CI[b])	Population Attributable Fraction % (95% CI[b])
Consumed any chicken meat***	87.2 (1,637/1,877)	79.1 (3,127/3,954)	1.6 (1.3-2.0)	32 (12-48)
Ate out (at food stand, restaurant, canteen, etc.)***	83.2 (1,656/1,990)	79.0 (3,270/4,141)	1.6 (1.3-2.0)	31 (19-39)
Prepared poultry meat (packaged) in household**	50.3 (908/1,805)	43.1 (1,681/3,898)	1.3 (1.1-1.5)	11 (3-16)
Prepared uncooked food and raw meat in household at the same time**	50.4 (930/1,846)	45.8 (1,770/3,867)	1.3 (1.1-1.5)	11 (3-17)
Used anti-acidic drug (PPI)***	21.2 (420/1,979)	8.0 (324/4,077)	1.9 (1.5-2.4)	10 (8-12)
Travelled abroad***	11.8 (237/2,001)	5.2 (213/4,135)	2.2 (1.6-2.9)	5 (4-6)
Had contact with poultry (animal)**	4.9 (96/1,954)	4.5 (181/4,063)	1.8 (1.3-2.6)	2 (2-3)
Consumed mostly vegetarian food*	2.1 (40/1,865)	4.3 (167/3,870)	0.5 (0.3-1.0)	-
Consumed (unpeeled) fruit***	62.4 (1,182/1,895)	73.0 (2,920/4,002)	0.6 (0.5-0.7)	-
Consumed lamb/mutton**	9.0 (163/1,821)	9.1 (359/3,969)	0.6 (0.5-0.8)	-
Consumed beef***	52.0 (911/1,752)	53.0 (2,043/3,855)	0.7 (0.6-0.8)	-
Had contact with dog**	29.0 (563/1,945)	32.6 (1,317/4,035)	0.8 (0.7-0.9)	-
Went swimming (in pool, lake, ocean etc.)**	18.3 (363/1,989)	24.9 (1,027/4,122)	0.8 (0.6-0.9)	-

[a]Adjusted odds ratios

[b]Confidence interval

*indicates *P*<0.05 **indicates *P*<0.01 ***indicates *P*<0.001

Supplementary Table S2. Factors positively associated (risk factors) and factors negatively associated with *Campylobacter* infections attributed to the source chicken (posterior probability *Pr*≥0.5) by source attribution using asymmetric island modelling. Case-control study, Germany, 2011-2014. The proportion of exposed cases and controls is based on the number of cases and controls with complete answers in univariable analysis (without adjustment for age group, sex, federal state). Adjusted odds ratios (aOR) were determined in multivariable logistic regression analysis (adjusted for age group, sex, federal state; 307 cases and 2,751 controls with complete answers for all variables in final model).

Risk Factor	Cases exposed % (n)	Controls exposed % (n)	aOR[a] (95% CI[b])	Population Attributable Fraction %
Consumed any chicken meat**	88.8 (395/445)	79,1 (2,967/3,753)	1.9 (1.3-2.7)	41
Ate out (at food stand, restaurant, canteen, etc.)*	82.0 (388/473)	78.6 (3,089/3,929)	1.5 (1.1-2.0)	25
Prepared poultry meat (packaged) in household**	55.7 (241/433)	43.8 (1,617/3,692)	1.6 (1.2-2.0)	21
Prepared uncooked food and raw meat in household at the same time**	54.3 (244/449)	45.8 (1,684/3,677)	1.4 (1.1-1.8)	17
Used anti-acidic drug (PPI)***	23.3 (110/473)	8.1 (315/3,869)	1.8 (1.3-2.5)	11
Consumed (unpeeled) fruit***	64.1 (284/443)	72,7 (2,757/3,794)	0.6 (0.5-0.8)	-
Consumed any duck meat**	15.7 (69/440)	14.9 (551/3,708)	0.6 (0.4-0.9)	-
Consumed any beef[c]	56.8 (237/417)	52.6 (1,923/3,654)	0.5 (0.3-0.9)	-
Had contact with dog**	24.8 (114/460)	32.8 (1,256/3,828)	0.7 (0.5-0.9)	-

[a]Adjusted odds ratio

[b]Confidence interval

[c]*P*=0.051

*indicates *P*<0.05 **indicates *P*<0.01 ***indicates *P*<0.001

Supplementary Table S3. MLST data and origin of animal and food isolates obtained during the study period (2011-2014) from samples taken in the study region through the FBI-Zoo-network (n=203; see Table 1, column 1). Isolates from pets, chickens, pigs, cattle and poultry other than chicken (ducks, geese, turkeys, quails) (n=188) were used for source attribution analysis, supplemented with MLST data from additional isolates obtained in Germany or in neighbouring countries.

Isolate Desig-nation	ST	CC	aspA	glnA	gltA	glyA	pgm	tkt	uncA	Origin	Animal Type	Animal Material	Food Material	Species	Source (For Source Attribution)
1	11	ST-45 complex	48	7	10	4	1	7	1	Animal	Dog			C. jejuni	Pet
2	19	ST-21 complex	2	1	5	3	2	1	5	Animal	Dog			C. jejuni	Pet
3	19	ST-21 complex	2	1	5	3	2	1	5	Animal	Dog			C. jejuni	Pet
4	19	ST-21 complex	2	1	5	3	2	1	5	Animal	Dog			C. jejuni	Pet
5	22	ST-22 complex	1	3	6	4	3	3	3	Animal	Cat			C. jejuni	Pet
7	50	ST-21 complex	2	1	12	3	2	1	5	Animal	Dog			C. jejuni	Pet
8	50	ST-21 complex	2	1	12	3	2	1	5	Animal	Dog			C. jejuni	Pet
9	50	ST-21 complex	2	1	12	3	2	1	5	Animal	Dog			C. jejuni	Pet
10	53	ST-21 complex	2	1	21	3	2	1	5	Animal	Cat			C. jejuni	Pet
11	53	ST-21 complex	2	1	21	3	2	1	5	Animal	Dog			C. jejuni	Pet
12	122	ST-206 complex	6	4	5	2	2	1	5	Animal	Cat			C. jejuni	Pet
21	464	ST-464 complex	24	2	2	2	10	3	1	Animal	Cat			C. jejuni	Pet
22	572	ST-206 complex	62	4	5	2	2	1	5	Animal	Dog			C. jejuni	Pet
33	658	ST-658 complex	2	4	2	4	19	3	6	Animal	Dog			C. jejuni	Pet
34	658	ST- 658 complex	2	4	2	4	19	3	6	Animal	Dog			C. jejuni	Pet
35	658	ST-658 complex	2	4	2	4	19	3	6	Animal	Dog			C. jejuni	Pet
57	2302	ST-828 complex	33	242	30	82	113	43	17	Animal	Dog			C. coli	Pet
58	2302	ST-828 complex	33	242	30	82	113	43	17	Animal	Dog			C. coli	Pet
63	3017	ST-828 complex	114	39	30	82	188	43	17	Animal	Cat			C. coli	Pet
68	6998		1	165	5	91	261	7	1	Animal	Cat			C. jejuni	Pet
69	6998		1	165	5	91	261	7	1	Animal	Cat			C. jejuni	Pet
13	122	ST-206 complex	6	4	5	2	2	1	5	Animal	Chicken	Feces		C. jejuni	Chicken
18	257	ST-257 complex	9	2	4	62	4	5	6	Animal	Chicken	Feces		C. jejuni	Chicken
51	2274		9	17	5	10	350	3	3	Animal	Chicken	Feces		C. jejuni	Chicken
52	2274		9	17	5	10	350	3	3	Animal	Chicken	Feces		C. jejuni	Chicken
53	2274		9	17	5	10	350	3	3	Animal	Chicken	Feces		C. jejuni	Chicken
54	2274		9	17	5	10	350	3	3	Animal	Chicken	Feces		C. jejuni	Chicken
55	2274		9	17	5	10	350	3	3	Animal	Chicken	Feces		C. jejuni	Chicken
56	2274		9	17	5	10	350	3	3	Animal	Chicken	Feces		C. jejuni	Chicken
66	5434	ST-828 complex	33	39	30	82	572	47	17	Animal	Chicken	Feces		C. coli	Chicken

Isolate Designation	ST	CC	aspA	glnA	gltA	glyA	pgm	tkt	uncA	Origin	Animal Type	Animal Material	Food Material	Species	Source (For Source Attribution)
67	5777	ST-828 complex	33	39	65	82	113	43	17	Animal	Chicken	Feces		C. coli	Chicken
77	7002		24	2	2	2	624	3	6	Animal	Chicken	Feces		C. jejuni	Chicken
FBI-08387	2100	ST-52 complex	9	25	2	10	22	3	8	Food	Chicken		Meat	C. jejuni	Chicken
FBI-08388	45	ST-45 complex	4	7	10	4	1	7	1	Food	Chicken		Meat	C. jejuni	Chicken
FBI-08389	45	ST-45 complex	4	7	10	4	1	7	1	Food	Chicken		Meat	C. jejuni	Chicken
FBI-08390	1701	ST-45 complex	4	7	10	4	1	51	1	Food	Chicken		Meat	C. jejuni	Chicken
FBI-08391	572	ST-206 complex	62	4	5	2	2	1	5	Food	Chicken		Meat	C. jejuni	Chicken
FBI-08392	50	ST-21 complex	2	1	12	3	2	1	5	Food	Chicken		Meat	C. jejuni	Chicken
FBI-08393	464	ST-464 complex	24	2	2	2	10	3	1	Food	Chicken		Meat	C. jejuni	Chicken
FBI-08394	354	ST-354 complex	8	10	2	2	11	12	6	Food	Chicken		Meat	C. jejuni	Chicken
FBI-08395	7386	ST-21 complex	2	504	5	3	2	25	5	Food	Chicken		Meat	C. jejuni	Chicken
FBI-08396	2314	ST-1034 complex	2	61	4	64	332	7	23	Food	Chicken		Meat	C. jejuni	Chicken
FBI-08397	7421		2	61	120	38	40	34	40	Food	Chicken		Meat	C. jejuni	Chicken
FBI-08398	904	ST-607 complex	24	2	5	53	23	3	1	Food	Chicken		Meat	C. jejuni	Chicken
FBI-08399	354	ST-354 complex	8	10	2	2	11	12	6	Food	Chicken		Meat	C. jejuni	Chicken
FBI-08400	45	ST-45 complex	4	7	10	4	1	7	1	Food	Chicken		Meat	C. jejuni	Chicken
FBI-08401	1301	ST-692 complex	2	115	57	26	127	29	35	Food	Chicken		Meat	C. jejuni	Chicken
FBI-08402	45	ST-45 complex	4	7	10	4	1	7	1	Food	Chicken		Meat	C. jejuni	Chicken
FBI-08403	44	ST-21 complex	8	1	6	3	2	1	1	Food	Chicken		Meat	C. jejuni	Chicken
FBI-08404	48	ST-48 complex	2	4	1	2	7	1	5	Food	Chicken		Meat	C. jejuni	Chicken
FBI-08405	7381	ST-1332 complex	2	1	4	28	721	25	58	Food	Chicken		Meat	C. jejuni	Chicken
FBI-08406	7381	ST-1332 complex	2	1	4	28	721	25	58	Food	Chicken		Meat	C. jejuni	Chicken
FBI-08407	48	ST-48 complex	2	4	1	2	7	1	5	Food	Chicken		Meat	C. jejuni	Chicken
FBI-08408	400	ST-353 complex	8	17	5	2	10	59	6	Food	Chicken		Meat	C. jejuni	Chicken
FBI-08409	324	ST-354 complex	57	17	2	2	11	12	6	Food	Chicken		Meat	C. jejuni	Chicken
FBI-08410	50	ST-21 complex	2	1	12	3	2	1	5	Food	Chicken		Meat	C. jejuni	Chicken
FBI-08411	7422		2	15	4	3	154	25	1	Food	Chicken		Meat	C. jejuni	Chicken
FBI-08412	3628	ST-443 complex	7	17	2	337	23	3	12	Food	Chicken		Meat	C. jejuni	Chicken
FBI-08413	7423	ST-283 complex	4	7	40	4	42	51	35	Food	Chicken		Meat	C. jejuni	Chicken
FBI-08414	860	ST-828 complex	33	39	30	79	113	47	17	Food	Chicken		Meat	C. coli	Chicken
FBI-08415	5150		292	66	30	82	113	206	17	Food	Chicken		Meat	C. coli	Chicken
FBI-08416	829	ST-828 complex	33	39	30	82	113	43	17	Food	Chicken		Meat	C. coli	Chicken
FBI-08417	1628	ST-828 complex	33	39	30	79	104	206	17	Food	Chicken		Meat	C. coli	Chicken
FBI-08418	7159	ST-828 complex	33	39	418	82	104	85	17	Food	Chicken		Meat	C. coli	Chicken
FBI-08419	7159	ST-828 complex	33	39	418	82	104	85	17	Food	Chicken		Meat	C. coli	Chicken
FBI-08420	7424	ST-828 complex	33	176	30	82	104	47	17	Food	Chicken		Meat	C. coli	Chicken
FBI-08421	825	ST-828 complex	33	39	30	82	113	47	17	Food	Chicken		Meat	C. coli	Chicken
FBI-08422	7389	ST-828 complex	33	39	30	82	189	43	425	Food	Chicken		Meat	C. coli	Chicken

Isolate Desig-nation	ST	CC	aspA	glnA	gltA	glyA	pgm	tkt	uncA	Origin	Animal Type	Animal Material	Food Material	Species	Source (For Source Attribution)
FBI-08423	828	ST-828 complex	33	39	30	82	104	43	17	Food	Chicken		Meat	C. coli	Chicken
FBI-08424	825	ST-828 complex	33	39	30	82	113	47	17	Food	Chicken		Meat	C. coli	Chicken
FBI-08425	7390	ST-828 complex	33	39	427	82	104	43	17	Food	Chicken		Meat	C. coli	Chicken
FBI-08426	1767	ST-828 complex	33	39	30	79	113	44	17	Food	Chicken		Meat	C. coli	Chicken
FBI-08427	5305	ST-828 complex	33	39	30	82	113	85	17	Food	Chicken		Meat	C. coli	Chicken
FBI-08556	829	ST-828 complex	33	39	30	82	113	43	17	Food	Chicken		unknown	C. coli	Chicken
FBI-08557	7431	ST-828 complex	33	195	30	82	104	47	17	Animal	Chicken	Feces		C. coli	Chicken
FBI-08558	5777	ST-828 complex	33	39	65	82	113	43	17	Animal	Chicken	Feces		C. coli	Chicken
FBI-08559	2183	ST-828 complex	33	39	30	79	113	43	41	Animal	Chicken	Feces		C. coli	Chicken
FBI-08560	829	ST-828 complex	33	39	30	82	113	43	17	Animal	Chicken	Feces		C. coli	Chicken
FBI-08564	1519	ST-21 complex	8	1	5	3	2	1	1	Food	Chicken		Meat	C. jejuni	Chicken
FBI-08565	1519	ST-21 complex	8	1	5	3	2	1	1	Food	Chicken		Meat	C. jejuni	Chicken
FBI-08568	257	ST-257 complex	9	2	4	62	4	5	6	Food	Chicken		other	C. jejuni	Chicken
FBI-08574	7394		18	33	78	18	724	575	16	Animal	Chicken	Feces		C. jejuni	Chicken
FBI-08575	5970		9	21	4	28	363	3	35	Animal	Chicken	Feces		C. jejuni	Chicken
FBI-08578	4800		275	84	5	10	119	178	26	Animal	Chicken	Feces		C. jejuni	Chicken
FBI-08579	305	ST-574 complex	9	53	2	10	11	3	3	Animal	Chicken			C. jejuni	Chicken
FBI-08580	5380	ST-828 complex	33	38	30	79	113	43	17	Animal	Chicken	Feces		C. coli	Chicken
FBI-08581	51	ST-443 complex	7	17	2	15	23	3	12	Food	Chicken		Meat	C. jejuni	Chicken
FBI-08582	50	ST-21 complex	2	1	12	3	2	1	5	Food	Chicken		Meat	C. jejuni	Chicken
FBI-08583	4899	ST-354 complex	9	2	2	2	11	59	6	Food	Chicken		Meat	C. jejuni	Chicken
FBI-08584	19	ST-21 complex	2	1	5	3	2	1	5	Food	Chicken		Meat	C. jejuni	Chicken
FBI-08585	22	ST-22 complex	1	3	6	4	3	3	3	Food	Chicken		Meat	C. jejuni	Chicken
FBI-08586	3766		22	28	4	28	363	3	35	Food	Chicken		Meat	C. jejuni	Chicken
FBI-08587	607	ST-607 complex	8	2	5	53	11	3	1	Food	Chicken		Meat	C. jejuni	Chicken
FBI-08588	2274		9	17	5	10	350	3	3	Food	Chicken		Meat	C. jejuni	Chicken
FBI-08589	400	ST-353 complex	8	17	5	2	10	59	6	Food	Chicken		Meat	C. jejuni	Chicken
FBI-08590	7054	ST-354 complex	8	10	2	2	19	12	6	Food	Chicken		Meat	C. jejuni	Chicken
FBI-08591	22	ST-22 complex	1	3	6	4	3	3	3	Food	Chicken		Meat	C. jejuni	Chicken
FBI-08592	2304		2	4	5	25	11	3	5	Food	Chicken		Meat	C. jejuni	Chicken
FBI-08593	50	ST-21 complex	2	1	12	3	2	1	5	Food	Chicken		Meat	C. jejuni	Chicken
FBI-08594	21	ST-21 complex	2	1	1	3	2	1	5	Food	Chicken		Meat	C. jejuni	Chicken
FBI-08595	1073	ST-354 complex	8	10	2	2	89	12	6	Food	Chicken		Meat	C. jejuni	Chicken
FBI-08596	22	ST-22 complex	1	3	6	4	3	3	3	Food	Chicken		Meat	C. jejuni	Chicken
FBI-08597	464	ST-464 complex	24	2	2	2	10	3	1	Food	Chicken		Meat	C. jejuni	Chicken
FBI-08598	50	ST-21 complex	2	1	12	3	2	1	5	Food	Chicken		Meat	C. jejuni	Chicken
FBI-08599	50	ST-21 complex	2	1	12	3	2	1	5	Food	Chicken		Meat	C. jejuni	Chicken
FBI-08600	2153		7	21	2	62	67	48	6	Food	Chicken		Meat	C. jejuni	Chicken

Isolate Desig-nation	ST	CC	aspA	glnA	gltA	glyA	pgm	tkt	uncA	Origin	Animal Type	Animal Material	Food Material	Species	Source (For Source Attribution)
FBI-08601	2220	ST-828 complex	33	38	30	82	337	43	17	Food	Chicken		Meat	C. coli	Chicken
FBI-08602	1595	ST-828 complex	33	38	30	79	104	43	17	Food	Chicken		Meat	C. coli	Chicken
FBI-08603	901	ST-828 complex	33	39	30	79	104	43	41	Food	Chicken		Meat	C. coli	Chicken
FBI-08604	832	ST-828 complex	33	39	30	79	113	43	17	Food	Chicken		Meat	C. coli	Chicken
FBI-08605	855	ST-828 complex	33	39	30	79	104	35	17	Food	Chicken		Meat	C. coli	Chicken
6	50	ST-21 complex	2	1	12	3	2	1	5	Animal	Quail			C. jejuni	Other Poultry
20	354	ST-354 complex	8	10	2	2	11	12	6	Animal	Quail			C. jejuni	Other Poultry
23	572	ST-206 complex	62	4	5	2	2	1	5	Animal	Quail			C. jejuni	Other Poultry
24	658	ST-658 complex	2	4	2	4	19	3	6	Animal	Quail			C. jejuni	Other Poultry
25	658	ST-658 complex	2	4	2	4	19	3	6	Animal	Quail			C. jejuni	Other Poultry
26	658	ST-658 complex	2	4	2	4	19	3	6	Animal	Quail			C. jejuni	Other Poultry
27	658	ST-658 complex	2	4	2	4	19	3	6	Animal	Quail			C. jejuni	Other Poultry
28	658	ST-658 complex	2	4	2	4	19	3	6	Animal	Quail			C. jejuni	Other Poultry
29	658	ST-658 complex	2	4	2	4	19	3	6	Animal	Quail			C. jejuni	Other Poultry
30	658	ST-658 complex	2	4	2	4	19	3	6	Animal	Quail			C. jejuni	Other Poultry
31	658	ST-658 complex	2	4	2	4	19	3	6	Animal	Quail			C. jejuni	Other Poultry
32	658	ST-658 complex	2	4	2	4	19	3	6	Animal	Quail			C. jejuni	Other Poultry
36	824	ST-257 complex	9	2	2	2	11	5	6	Animal	Quail			C. jejuni	Other Poultry
37	829	ST-828 complex	33	39	30	82	113	43	17	Animal	Quail			C. coli	Other Poultry
45	1519	ST-21 complex	8	1	5	3	2	1	1	Animal	Quail			C. jejuni	Other Poultry
46	1586	ST-828 complex	33	176	30	82	113	43	17	Animal	Quail			C. coli	Other Poultry
62	2929	ST-21 complex	2	1	12	3	2	1	1	Animal	Quail			C. jejuni	Other Poultry
64	5172	ST-828 complex	33	195	30	79	113	43	17	Animal	Quail			C. coli	Other Poultry
65	5333	ST-45 complex	2	7	10	4	1	51	1	Animal	Quail			C. jejuni	Other Poultry
70	6999	ST-658 complex	2	4	27	4	19	3	6	Animal	Quail			C. jejuni	Other Poultry
71	7000		7	21	2	62	525	48	1	Animal	Quail			C. jejuni	Other Poultry
72	7000		7	21	2	62	525	48	1	Animal	Quail			C. jejuni	Other Poultry
73	7000		7	21	2	62	525	48	1	Animal	Quail			C. jejuni	Other Poultry
74	7000		7	21	2	62	525	48	1	Animal	Quail			C. jejuni	Other Poultry
75	7000		7	21	2	62	525	48	1	Animal	Quail			C. jejuni	Other Poultry
76	7001		7	21	2	62	525	48	5	Animal	Quail			C. jejuni	Other Poultry
FBI-08562	50	ST-21 complex	2	1	12	3	2	1	5	Food	Duck		Meat	C. jejuni	Other Poultry
FBI-08563	3574	ST-21 complex	2	1	12	3	2	1	23	Food	Duck		Meat	C. jejuni	Other Poultry
FBI-08561	475	ST-48 complex	2	4	1	4	19	62	5	Food	Turkey		Meat	C. jejuni	Other Poultry
38	854	ST-828 complex	33	38	30	82	104	43	17	Animal	Pig			C. coli	Pig
39	854	ST-828 complex	33	38	30	82	104	43	17	Animal	Pig			C. coli	Pig
40	854	ST-828 complex	33	38	30	82	104	43	17	Animal	Pig			C. coli	Pig
41	854	ST-828 complex	33	38	30	82	104	43	17	Animal	Pig			C. coli	Pig
42	854	ST-828 complex	33	38	30	82	104	43	17	Animal	Pig			C. coli	Pig

Isolate Desig-nation	ST	CC	aspA	glnA	gltA	glyA	pgm	tkt	uncA	Origin	Animal Type	Animal Material	Food Material	Species	Source (For Source Attribution)
43	854	ST-828 complex	33	38	30	82	104	43	17	Animal	Pig			C. coli	Pig
44	1430		32	153	30	82	104	44	36	Animal	Pig			C. coli	Pig
59	2642	ST-828 complex	33	38	30	79	104	35	17	Animal	Pig			C. coli	Pig
60	2705	ST-828 complex	33	153	44	82	104	43	36	Animal	Pig			C. coli	Pig
61	2814	ST-828 complex	32	38	30	82	104	43	36	Animal	Pig			C. coli	Pig
78	7003		32	153	412	82	104	44	36	Animal	Pig			C. coli	Pig
79	7003		32	153	412	82	104	44	36	Animal	Pig			C. coli	Pig
80	7004		32	153	412	82	104	44	17	Animal	Pig			C. coli	Pig
81	7005	ST-828 complex	33	39	30	161	104	117	17	Animal	Pig			C. coli	Pig
82	7005	ST-828 complex	33	39	30	161	104	117	17	Animal	Pig			C. coli	Pig
83	7005	ST-828 complex	33	39	30	161	104	117	17	Animal	Pig			C. coli	Pig
84	7005	ST-828 complex	33	39	30	161	104	117	17	Animal	Pig			C. coli	Pig
85	7005	ST-828 complex	33	39	30	161	104	117	17	Animal	Pig			C. coli	Pig
86	7005	ST-828 complex	33	39	30	161	104	117	17	Animal	Pig			C. coli	Pig
87	7006	ST-828 complex	33	39	30	161	104	44	17	Animal	Pig			C. coli	Pig
FBI-08527	7426	ST-828 complex	33	38	30	78	104	43	17	Animal	Pig	Caecum		C. coli	Pig
FBI-08528	1117	ST-828 complex	33	39	30	78	104	35	17	Animal	Pig	Caecum		C. coli	Pig
FBI-08529	1156	ST-828 complex	33	39	30	79	104	64	17	Animal	Pig	Caecum		C. coli	Pig
FBI-08530	4950	ST-828 complex	33	38	30	82	113	332	17	Animal	Pig	Caecum		C. coli	Pig
FBI-08531	1156	ST-828 complex	33	39	30	79	104	64	17	Animal	Pig	Caecum		C. coli	Pig
FBI-08532	1117	ST-828 complex	33	39	30	78	104	35	17	Animal	Pig	Surface carcass		C. coli	Pig
FBI-08533	1166	ST-828 complex	33	39	30	79	104	44	17	Animal	Pig	Caecum		C. coli	Pig
FBI-08534	1108	ST-828 complex	33	39	30	78	113	43	17	Animal	Pig	Caecum		C. coli	Pig
FBI-08535	828	ST-828 complex	33	39	30	82	104	43	17	Animal	Pig	Feces		C. coli	Pig
FBI-08536	1554	ST-828 complex	33	66	30	82	118	43	17	Animal	Pig	Feces		C. coli	Pig
FBI-08537	828	ST-828 complex	33	39	30	82	104	43	17	Animal	Pig	Feces		C. coli	Pig
FBI-08538	854	ST-828 complex	33	38	30	82	104	43	17	Animal	Pig	Feces		C. coli	Pig
FBI-08539	1103	ST-828 complex	33	39	30	174	104	35	17	Animal	Pig	Feces		C. coli	Pig
FBI-08540	4087		33	38	44	82	104	44	68	Animal	Pig	Feces		C. coli	Pig
FBI-08541	1068	ST-828 complex	33	39	30	78	104	43	17	Animal	Pig	Feces		C. coli	Pig
FBI-08542	828	ST-828 complex	33	39	30	82	104	43	17	Animal	Pig	Feces		C. coli	Pig
FBI-08544	7392	ST-828 complex	33	39	30	573	118	35	17	Animal	Pig	Caecum		C. coli	Pig
FBI-08545	1103	ST-828 complex	33	39	30	174	104	35	17	Animal	Pig	Caecum		C. coli	Pig
FBI-08546	4087		33	38	44	82	104	44	68	Animal	Pig	Caecum		C. coli	Pig
FBI-08547	828	ST-828 complex	33	39	30	82	104	43	17	Animal	Pig	Caecum		C. coli	Pig
FBI-08548	1554	ST-828 complex	33	66	30	82	118	43	17	Animal	Pig	Caecum		C. coli	Pig
FBI-08549	7427	ST-828 complex	32	39	30	174	104	43	36	Animal	Pig	Feces		C. coli	Pig
FBI-08550	7428	ST-828 complex	33	38	30	82	189	64	17	Animal	Pig	Caecum		C. coli	Pig
FBI-08551	828	ST-828 complex	33	39	30	82	104	43	17	Animal	Pig	Caecum		C. coli	Pig

Isolate Desig-nation	ST	CC	aspA	glnA	gltA	glyA	pgm	tkt	uncA	Origin	Animal Type	Animal Material	Food Material	Species	Source (For Source Attribution)
FBI-08552	1096	ST-828 complex	33	38	30	82	104	35	17	Animal	Pig	Caecum		C. coli	Pig
FBI-08553	7429	ST-828 complex	302	39	30	82	104	47	17	Animal	Pig	Caecum		C. coli	Pig
FBI-08554	7428	ST-828 complex	33	38	30	82	189	64	17	Animal	Pig	Caecum		C. coli	Pig
FBI-08577	122	ST-206 complex	6	4	5	2	2	1	5	Food	Cattle		Raw milk	C. jejuni	Cattle
14	220	ST-179 complex	1	6	29	2	40	32	3	Animal	Pigeon			C. jejuni	Not considered
15	220	ST-179 complex	1	6	29	2	40	32	3	Animal	Pigeon			C. jejuni	Not considered
16	220	ST-179 complex	1	6	29	2	40	32	3	Animal	Pigeon			C. jejuni	Not considered
17	220	ST-179 complex	1	6	29	2	40	32	3	Animal	Pigeon			C. jejuni	Not considered
19	334	ST-45 complex	4	7	40	4	42	7	1	Animal	Pigeon			C. jejuni	Not considered
47	1962		55	172	21	49	125	83	51	Animal	Pigeon			C. jejuni	Not considered
48	1962		55	172	21	49	125	83	51	Animal	Pigeon			C. jejuni	Not considered
49	1962		55	172	21	49	125	83	51	Animal	Pigeon			C. jejuni	Not considered
50	1962		55	172	21	49	125	83	51	Animal	Pigeon			C. jejuni	Not considered
FBI-08570	220	ST-179 complex	1	6	29	2	40	32	3	Animal	Pigeon (aviary bird)	Feces		C. jejuni	Not considered
FBI-08571	4447	ST-179 complex	1	31	29	176	40	32	3	Animal	Pigeon (aviary bird)	Feces		C. jejuni	Not considered
FBI-08573	4447	ST-179 complex	1	31	29	176	40	32	3	Animal	Pigeon (aviary bird)	Feces		C. jejuni	Not considered
FBI-08555	7393		148	90	126	226	723	574	426	Food	Common mussel			C. coli	Not considered
FBI-08566	5053		1	2	95	62	472	400	147	Food	Common mussel			C. jejuni	Not considered
FBI-08569	3573		7	172	21	49	125	224	51	Animal	Wild bird			C. jejuni	Not considered

Supplementary Table S4. MLST data and relative posterior source probability of 613 human *Campylobacter* isolates obtained during the study period (2011-2014), according to sequence type. As putative sources we considered pet, chicken, cattle, pig, and poultry other than chicken (duck, goose, turkey, quail: "other poultry"). Source probabilities were assigned using asymmetric island modelling.

	MLST DATA									POSTERIOR ASSIGNMENT SOURCE PROBABILITIES				
Number of human isolates	Sequence Type (ST)	Clonal Complex (CC)	aspA	glnA	gltA	glyA	pgm	tkt	uncA	Pet	Chicken	Cattle	Pig	Other Poultry
1	5	ST-353 complex	7	2	5	2	10	3	6	0.140358	0.792519	0.004921	0.000065	0.062137
6	19	ST-21 complex	2	1	5	3	2	1	5	0.329778	0.535305	0.075741	0.001287	0.057890
20	21	ST-21 complex	2	1	1	3	2	1	5	0.206268	0.659486	0.066991	0.001201	0.066053
6	22	ST-22 complex	1	3	6	4	3	3	3	0.316581	0.622470	0.022396	0.000743	0.037809
1	25	ST-45 complex	4	7	10	1	1	7	1	0.199498	0.646314	0.004851	0.000365	0.148971
1	42	ST-42 complex	1	2	3	4	5	9	3	0.361405	0.514092	0.083063	0.001401	0.040040
6	44	ST-21 complex	8	1	6	3	2	1	1	0.044326	0.836094	0.002239	0.000707	0.116634
15	45	ST-45 complex	4	7	10	4	1	7	1	0.214536	0.689252	0.009782	0.000746	0.085684
3	46	ST-206 complex	2	21	5	3	2	1	5	0.049799	0.896103	0.002500	0.000469	0.051129
13	48	ST-48 complex	2	4	1	2	7	1	5	0.270188	0.661681	0.018949	0.000711	0.048471
1	49	ST-49 complex	3	1	5	17	11	11	6	0.050397	0.854069	0.001084	0.000248	0.094202
91	50	ST-21 complex	2	1	12	3	2	1	5	0.160770	0.738177	0.020941	0.000787	0.079325
3	51	ST-443 complex	7	17	2	15	23	3	12	0.164981	0.747140	0.003920	0.000664	0.083295
2	52	ST-52 complex	9	25	2	10	22	3	6	0.257587	0.696787	0.005297	0.000557	0.039771
3	53	ST-21 complex	2	1	21	3	2	1	5	0.215735	0.684886	0.020337	0.000797	0.078244
2	61	ST-61 complex	1	4	2	2	6	3	17	0.308651	0.550106	0.096325	0.001607	0.043311
29	122	ST-206 complex	6	4	5	2	2	1	5	0.210124	0.738521	0.008452	0.000568	0.042335
1	131	not assigned	7	2	6	10	10	37	1	0.048351	0.899379	0.002103	0.000480	0.049687
4	137	ST-45 complex	4	7	10	4	42	7	1	0.373810	0.546239	0.007389	0.000732	0.071830
1	206	ST-206 complex	2	21	5	37	2	1	5	0.023278	0.490408	0.002946	0.002246	0.481122
1	233	ST-45 complex	2	7	10	4	1	7	1	0.569782	0.311637	0.058884	0.000983	0.058714
29	257	ST-257 complex	9	2	4	62	4	5	6	0.090015	0.847299	0.007854	0.000570	0.054262
7	267	ST-283 complex	4	7	40	4	42	51	1	0.132395	0.779389	0.016795	0.000735	0.070687
1	273	ST-206 complex	2	21	5	37	60	1	5	0.021486	0.416450	0.001156	0.000922	0.559986
1	305	ST-574 complex	9	53	2	10	11	3	3	0.302236	0.653541	0.006043	0.000575	0.037605
1	324	ST-354 complex	57	17	2	2	11	12	6	0.048462	0.899185	0.002110	0.000481	0.049762
4	353	ST-353 complex	7	17	5	2	10	3	6	0.277248	0.677578	0.005620	0.000562	0.038993
27	354	ST-354 complex	8	10	2	2	11	12	6	0.044215	0.831003	0.002185	0.000741	0.121856
7	356	ST-353 complex	14	17	5	2	11	3	6	0.048365	0.899256	0.002106	0.000479	0.049794
5	400	ST-353 complex	8	17	5	2	10	59	6	0.257317	0.697171	0.005296	0.000557	0.039658

	MLST DATA									POSTERIOR ASSIGNMENT SOURCE PROBABILITIES				
Number of human isolates	Sequence Type (ST)	Clonal Complex (CC)	aspA	glnA	gltA	glyA	pgm	tkt	uncA	Pet	Chicken	Cattle	Pig	Other Poultry
1	403	ST-403 complex	10	27	16	19	10	5	7	0.792751	0.174119	0.015732	0.000982	0.016416
4	429	ST-48 complex	7	4	5	2	11	1	5	0.066336	0.807177	0.001223	0.000002	0.125261
2	441	not assigned	7	1	2	83	2	3	6	0.597225	0.355858	0.020859	0.000798	0.025261
4	443	ST-443 complex	24	17	2	15	23	3	12	0.036646	0.710168	0.002341	0.001235	0.249611
2	446	ST-446 complex	47	55	5	10	11	68	8	0.049357	0.829468	0.000789	0.000039	0.120347
23	464	ST-464 complex	24	2	2	2	10	3	1	0.076274	0.852996	0.002554	0.000555	0.067621
2	523	ST-658 complex	2	4	1	93	11	3	6	0.048662	0.898706	0.002145	0.000478	0.050009
1	524	ST-353 complex	8	17	5	2	10	3	1	0.161769	0.669112	0.002321	0.000005	0.166793
1	534	not assigned	7	78	42	95	106	12	8	0.048522	0.899507	0.002110	0.000486	0.049376
1	538	ST-45 complex	4	7	10	4	42	25	1	0.090327	0.741363	0.001306	0.000046	0.166959
44	572	ST-206 complex	62	4	5	2	2	1	5	0.168130	0.767682	0.009495	0.000603	0.054091
1	574	ST-574 complex	7	53	2	10	11	3	3	0.225102	0.704676	0.002084	0.000037	0.068101
1	577	ST-21 complex	2	4	1	3	2	1	5	0.304841	0.407882	0.212623	0.002043	0.072611
4	583	ST-45 complex	4	7	10	4	42	51	1	0.050031	0.896641	0.002202	0.000474	0.050652
1	587	ST-362 complex	1	2	42	4	90	25	8	0.048603	0.899218	0.002124	0.000483	0.049572
1	607	ST-607 complex	8	2	5	53	11	3	1	0.257076	0.697291	0.005292	0.000555	0.039786
1	657	ST-658 complex	73	4	2	4	19	3	6	0.345922	0.293993	0.004153	0.000633	0.355299
4	658	ST-658 complex	2	4	2	4	19	3	6	0.423848	0.312928	0.008916	0.001464	0.252843
1	677	ST-677 complex	10	81	50	99	120	76	52	0.496654	0.464609	0.009455	0.000669	0.028613
1	768	not assigned	7	2	5	72	22	102	6	0.050105	0.786872	0.000193	0.000008	0.162822
11	824	ST-257 complex	9	2	2	2	11	5	6	0.040268	0.774136	0.002252	0.000966	0.182379
7	825	ST-828 complex	33	39	30	82	113	47	17	0.049465	0.878883	0.005626	0.001482	0.064543
3	827	ST-828 complex	33	39	30	82	104	56	17	0.049084	0.862593	0.006228	0.000918	0.081176
1	828	ST-828 complex	33	39	30	82	104	43	17	0.035785	0.672856	0.002734	0.134501	0.154124
3	829	ST-828 complex	33	39	30	82	113	43	17	0.043909	0.848607	0.002096	0.003577	0.101811
2	832	ST-828 complex	33	39	30	79	113	43	17	0.040163	0.787417	0.002217	0.012774	0.157428
1	842	ST-21 complex	2	1	42	3	2	1	5	0.208031	0.651472	0.070483	0.000071	0.069942
1	854	ST-828 complex	33	38	30	82	104	43	17	0.037591	0.596634	0.003860	0.329833	0.032083
2	860	ST-828 complex	33	39	30	79	113	47	17	0.048492	0.816512	0.008848	0.001130	0.125018
2	861	ST-21 complex	2	1	42	3	148	1	5	0.117573	0.746329	0.083319	0.001317	0.051463
1	881	not assigned	9	17	52	10	10	3	1	0.495388	0.465613	0.009421	0.000661	0.028917
4	883	ST-21 complex	2	17	2	3	2	1	5	0.324001	0.600077	0.006721	0.000684	0.068517
1	887	ST-828 complex	33	38	30	82	104	85	68	0.028420	0.239128	0.006623	0.715459	0.010370
1	890	ST-828 complex	33	38	30	82	104	35	36	0.018111	0.180024	0.004219	0.788520	0.009126
1	899	ST-828 complex	33	39	30	82	113	35	17	0.023137	0.369828	0.005159	0.548901	0.052974
7	904	ST-607 complex	24	2	5	53	23	3	1	0.259629	0.623449	0.005501	0.000816	0.110605
1	918	ST-48 complex	2	4	1	4	19	1	5	0.743772	0.182867	0.050322	0.001108	0.021931
1	977	ST-1034 complex	22	61	4	64	74	25	23	0.040258	0.774232	0.002250	0.000980	0.182279

	MLST DATA									POSTERIOR ASSIGNMENT SOURCE PROBABILITIES				
Number of human isolates	Sequence Type (ST)	Clonal Complex (CC)	aspA	glnA	gltA	glyA	pgm	tkt	uncA	Pet	Chicken	Cattle	Pig	Other Poultry
1	986	not assigned	91	2	42	4	169	9	8	0.087869	0.593405	0.004881	0.000024	0.313822
2	990	ST-257 complex	9	2	4	62	4	133	6	0.040153	0.774339	0.002245	0.000972	0.182290
1	991	ST-692 complex	37	52	57	26	107	29	23	0.048515	0.899477	0.002111	0.000486	0.049411
1	1009	not assigned	32	66	66	82	163	43	17	0.000015	0.002075	0.000005	0.997439	0.000465
1	1016	ST-828 complex	33	38	30	82	118	43	17	0.038054	0.598936	0.003934	0.327162	0.031914
1	1038	ST-354 complex	8	10	5	2	11	12	6	0.026593	0.819756	0.001551	0.000041	0.152060
3	1044	ST-658 complex	2	10	2	4	19	3	6	0.496140	0.463013	0.009514	0.000655	0.030677
3	1056	ST-828 complex	33	39	30	82	104	43	36	0.015856	0.199322	0.003609	0.766891	0.014321
1	1058	ST-828 complex	33	39	30	82	104	35	17	0.028077	0.570603	0.002339	0.204179	0.194802
1	1073	ST-354 complex	8	10	2	2	89	12	6	0.228332	0.699518	0.010383	0.000673	0.061093
1	1122	ST-828 complex	33	153	30	82	118	43	36	0.000556	0.025787	0.000242	0.972192	0.001222
1	1123	not assigned	53	38	44	82	118	35	36	0.000716	0.025370	0.000354	0.970780	0.002779
1	1147	not assigned	53	38	30	82	118	44	36	0.029026	0.242359	0.006762	0.711418	0.010434
1	1181	ST-828 complex	33	39	66	174	104	43	41	0.022386	0.824810	0.001719	0.060453	0.090631
2	1301	ST-692 complex	2	115	57	26	127	29	35	0.036331	0.709746	0.002376	0.001250	0.250297
1	1348	ST-61 complex	108	4	2	146	6	3	6	0.023380	0.491641	0.002886	0.002284	0.479809
1	1409	not assigned	2	15	4	3	154	51	35	0.025185	0.903778	0.001311	0.000028	0.069698
2	1417	ST-828 complex	33	39	44	82	104	35	36	0.024721	0.209180	0.006253	0.750235	0.009611
2	1450	not assigned	53	39	44	82	104	35	36	0.001816	0.099762	0.000421	0.892764	0.005238
4	1519	ST-21 complex	8	1	5	3	2	1	1	0.042037	0.804861	0.002261	0.000821	0.150020
1	1528	ST-607 complex	8	2	5	68	11	5	1	0.103036	0.830198	0.001658	0.000004	0.065104
1	1545	ST-828 complex	33	39	30	82	118	43	17	0.040418	0.729121	0.002721	0.186664	0.041075
1	1549	ST-828 complex	33	38	30	82	113	44	17	0.004058	0.291203	0.000527	0.687058	0.017153
2	1571	ST-828 complex	32	39	30	82	104	43	36	0.024076	0.206388	0.005621	0.754739	0.009176
1	1585	ST-828 complex	33	39	30	82	189	43	17	0.045674	0.883283	0.002003	0.015007	0.054033
2	1595	ST-828 complex	33	38	30	79	104	43	17	0.044667	0.832443	0.002402	0.073237	0.047251
3	1628	ST-828 complex	33	39	30	79	104	206	17	0.054122	0.842650	0.012629	0.000884	0.089716
1	1707	ST-607 complex	9	2	5	2	11	3	1	0.047929	0.899422	0.002088	0.000470	0.050091
1	1796	ST-257 complex	9	2	4	62	10	5	6	0.085913	0.832320	0.004153	0.000737	0.076877
1	1911	not assigned	7	84	5	10	119	178	26	0.048149	0.897233	0.002092	0.000480	0.052047
2	1943	ST-21 complex	2	1	1	3	2	226	5	0.048675	0.898413	0.002218	0.000474	0.050220
2	1947	ST-22 complex	1	94	6	4	3	3	3	0.497042	0.463991	0.009519	0.000660	0.028789
2	2036	ST-353 complex	7	17	52	10	11	3	6	0.012970	0.898188	0.000192	0.000001	0.088649
1	2057	ST-21 complex	2	1	4	3	2	1	5	0.063584	0.749595	0.016222	0.000068	0.170531
1	2076	ST-353 complex	7	17	5	2	86	232	1	0.138742	0.651039	0.000662	0.000009	0.209548
3	2079	ST-48 complex	14	4	1	4	19	1	5	0.420365	0.504813	0.018435	0.000110	0.056277
1	2083	not assigned	8	2	2	212	309	253	147	0.111421	0.447491	0.000863	0.000015	0.440209
2	2100	ST-52 complex	9	25	2	10	22	3	8	0.048507	0.899211	0.002110	0.000481	0.049691

	MLST DATA									POSTERIOR ASSIGNMENT SOURCE PROBABILITIES				
Number of human isolates	Sequence Type (ST)	Clonal Complex (CC)	aspA	glnA	gltA	glyA	pgm	tkt	uncA	Pet	Chicken	Cattle	Pig	Other Poultry
1	2122	ST-353 complex	7	17	5	2	86	3	1	0.135191	0.734584	0.001051	0.000016	0.129158
1	2123	ST-362 complex	1	2	42	4	2	25	8	0.046231	0.871421	0.004509	0.000053	0.077787
1	2130	not assigned	43	194	2	213	19	231	148	0.223885	0.248594	0.001526	0.000079	0.525916
1	2133	not assigned	55	21	2	71	11	37	3	0.009973	0.769138	0.000032	0.000000	0.220857
1	2209	ST-179 complex	1	6	29	176	40	32	3	0.113046	0.362203	0.000554	0.000000	0.524197
2	2254	ST-257 complex	8	2	4	62	4	5	6	0.048102	0.895292	0.002156	0.000449	0.054002
13	2274	not assigned	9	17	5	10	350	3	3	0.048336	0.899458	0.002106	0.000480	0.049621
2	2275	ST-52 complex	9	2	1	10	22	3	6	0.204158	0.698226	0.016467	0.000004	0.081146
3	2304	not assigned	2	4	5	25	11	3	5	0.050105	0.897275	0.002178	0.000475	0.049968
2	2497	ST-22 complex	1	3	6	294	3	3	3	0.299963	0.615387	0.011804	0.000384	0.072462
1	2508	ST-828 complex	33	38	30	82	104	35	68	0.001292	0.061194	0.000358	0.931553	0.005604
1	2642	ST-828 complex	33	38	30	79	104	35	17	0.040280	0.737011	0.002707	0.176366	0.043636
1	2801	ST-353 complex	7	112	5	62	11	3	6	0.042899	0.861759	0.001304	0.000051	0.093987
1	2814	ST-828 complex	32	38	30	82	104	43	36	0.019271	0.187373	0.004465	0.779746	0.009145
1	2897	not assigned	7	71	5	303	11	67	1	0.048384	0.899426	0.002102	0.000481	0.049608
1	3017	ST-828 complex	114	39	30	82	188	43	17	0.368205	0.588922	0.007156	0.000724	0.034993
1	3335	ST-206 complex	62	4	5	10	2	1	5	0.080468	0.845073	0.002632	0.000032	0.071795
1	3339	ST-828 complex	33	39	30	82	414	43	17	0.047489	0.899253	0.002073	0.000594	0.050591
1	3628	ST-443 complex	7	17	2	337	23	3	12	0.048493	0.899143	0.002109	0.000482	0.049774
1	3633	ST-21 complex	2	1	1	5	2	343	5	0.658567	0.240952	0.041135	0.000050	0.059296
3	3720	ST-49 complex	3	1	5	10	11	11	6	0.048394	0.899313	0.002119	0.000481	0.049693
1	4086	ST-828 complex	33	39	44	82	104	44	68	0.026468	0.224105	0.006177	0.733440	0.009810
3	4709	ST-828 complex	33	39	30	296	104	85	17	0.047479	0.899307	0.002074	0.000590	0.050550
1	4713	ST-607 complex	166	17	5	10	11	3	1	0.030309	0.764600	0.000225	0.000001	0.204865
1	4735	ST-21 complex	2	1	12	407	2	1	5	0.153805	0.695091	0.010345	0.000389	0.140370
2	4801	not assigned	7	21	2	62	525	48	6	0.047612	0.886170	0.002075	0.000474	0.063669
1	4811	not assigned	6	4	52	2	89	282	5	0.062157	0.850905	0.003635	0.000000	0.083303
1	5018	ST-21 complex	2	1	1	3	492	1	5	0.329500	0.302257	0.305320	0.004113	0.058810
4	5150	not assigned	292	66	30	82	113	206	17	0.035803	0.708775	0.002330	0.001422	0.251670
1	5203	not assigned	2	15	4	28	20	34	35	0.009437	0.875968	0.000507	0.000030	0.114059
1	5306	ST-828 complex	33	38	30	82	104	468	17	0.027452	0.231793	0.006407	0.724247	0.010102
1	5326	not assigned	188	313	5	2	89	5	6	0.072431	0.582167	0.000927	0.000002	0.344472
1	5759	not assigned	22	28	4	295	372	99	35	0.001783	0.169818	0.000085	0.000048	0.828266
1	5776	not assigned	32	39	30	81	118	44	36	0.000245	0.004826	0.000174	0.994522	0.000233
1	6175	ST-21 complex	2	1	5	10	608	1	5	0.163118	0.672439	0.011194	0.000035	0.153214
1	6409	ST-1034 complex	2	15	4	3	154	25	23	0.008954	0.861326	0.000317	0.000031	0.129372
2	6461	ST-353 complex	2	17	5	2	10	59	6	0.281230	0.634880	0.012270	0.000049	0.071571
1	6557	not assigned	76	22	165	98	146	254	16	0.028445	0.650297	0.000048	0.000003	0.321206

	MLST DATA									POSTERIOR ASSIGNMENT SOURCE PROBABILITIES				
Number of human isolates	Sequence Type (ST)	Clonal Complex (CC)	aspA	glnA	gltA	glyA	pgm	tkt	uncA	Pet	Chicken	Cattle	Pig	Other Poultry
1	6813	not assigned	2	17	52	10	11	3	6	0.251598	0.559242	0.003838	0.000000	0.185321
1	6909	ST-574 complex	9	2	2	10	11	3	3	0.208607	0.726205	0.006692	0.000039	0.058458
3	7000	not assigned	7	21	2	62	525	48	1	0.027797	0.570244	0.002636	0.001880	0.397443
1	7143	ST-21 complex	2	1	1	563	2	309	5	0.192449	0.586162	0.020591	0.000338	0.200460
1	7159	ST-828 complex	33	39	418	82	104	85	17	0.047681	0.899375	0.002082	0.000587	0.050276
1	7214	ST-828 complex	33	39	103	82	104	47	17	0.006529	0.681348	0.000329	0.004860	0.306934
1	7377	ST-828 complex	33	38	30	82	113	571	17	0.045465	0.733602	0.001920	0.136081	0.082932
1	7378	not assigned	7	17	2	572	86	232	1	0.104912	0.491112	0.000348	0.000006	0.403621
1	7379	ST-1034 complex	2	61	4	64	332	7	422	0.050495	0.853720	0.001087	0.000249	0.094450
1	7380	ST-21 complex	2	501	12	3	2	1	5	0.152735	0.696640	0.010245	0.000388	0.139992
1	7381	ST-1332 complex	2	1	4	28	721	25	58	0.048502	0.898980	0.002142	0.000481	0.049894
1	7382	ST-828 complex	33	502	30	240	722	43	17	0.013095	0.555618	0.000154	0.221545	0.209587
1	7383	ST-21 complex	2	1	12	3	2	573	5	0.153742	0.695213	0.010337	0.000390	0.140317
1	7384	ST-52 complex	9	503	2	10	22	3	6	0.246085	0.674932	0.002807	0.000286	0.075890
1	7385	not assigned	32	38	30	82	104	43	424	0.030014	0.290704	0.003633	0.652291	0.023358
1	7387	ST-353 complex	8	7	5	2	10	59	6	0.256998	0.671461	0.004511	0.000039	0.066992
1	7388	not assigned	22	28	2	82	363	3	35	0.015834	0.824610	0.001227	0.000871	0.157459
1	7391	not assigned	365	71	5	62	2	1	1	0.008003	0.680535	0.000665	0.000001	0.310796
1	7395	ST-828 complex	33	39	30	79	104	117	36	0.000201	0.058698	0.000050	0.929697	0.011353
1	7396	ST-353 complex	2	17	52	10	10	3	6	0.469658	0.411864	0.011830	0.000007	0.106641
3	7397	ST-21 complex	2	1	1	3	141	1	5	0.045348	0.750818	0.011925	0.000089	0.191820
1	7399	not assigned	32	39	30	81	118	43	36	0.000009	0.000599	0.000006	0.999325	0.000062
1	7401	not assigned	2	21	5	62	67	3	216	0.035434	0.579253	0.004749	0.000096	0.380468
1	7402	ST-354 complex	2	10	12	2	11	12	6	0.030784	0.641042	0.005211	0.000004	0.322959
1	7403	ST-45 complex	4	7	10	526	42	7	1	0.337890	0.530058	0.003839	0.000363	0.127850
1	7404	not assigned	9	17	5	10	127	3	3	0.009027	0.868463	0.000311	0.000030	0.122169
2	7405	ST-354 complex	8	7	5	2	11	59	6	0.151381	0.738266	0.001677	0.000003	0.108673
1	7406	not assigned	32	39	30	79	118	117	17	0.000006	0.003198	0.000004	0.995725	0.001067
1	7408	ST-49 complex	3	1	5	82	104	11	6	0.002995	0.802379	0.000328	0.085149	0.109150
1	7409	ST-49 complex	3	1	5	17	104	11	6	0.011649	0.842172	0.000387	0.003476	0.142316
1	7410	not assigned	62	4	44	82	104	1	36	0.074626	0.510491	0.047767	0.292885	0.074232
1	7411	ST-828 complex	32	39	30	82	113	43	36	0.001753	0.054805	0.000532	0.937674	0.005235
2	7412	ST-828 complex	33	39	30	82	231	43	17	0.009065	0.872239	0.000353	0.002182	0.116161
1	7413	not assigned	7	4	2	2	19	1	26	0.009262	0.879858	0.000316	0.000028	0.110536
1	7414	ST-828 complex	33	1	30	82	113	43	17	0.106264	0.730077	0.008545	0.000905	0.154209
1	7415	ST-661 complex	2	28	4	406	90	34	1	0.001579	0.518909	0.000105	0.000000	0.479408
1	7416	ST-353 complex	7	2	12	299	10	3	6	0.033365	0.874078	0.002475	0.000035	0.090048
1	7417	not assigned	22	61	4	28	363	99	35	0.002715	0.311636	0.000277	0.000098	0.685274

	MLST DATA									POSTERIOR ASSIGNMENT SOURCE PROBABILITIES				
**Number of human isolates**	**Sequence Type (ST)**	**Clonal Complex (CC)**	**aspA**	**glnA**	**gltA**	**glyA**	**pgm**	**tkt**	**uncA**	**Pet**	**Chicken**	**Cattle**	**Pig**	**Other Poultry**
1	7419	ST-21 complex	2	1	12	79	2	1	5	0.032982	0.767136	0.005058	0.001130	0.193694
1	7420	not assigned	27	255	105	18	25	160	16	0.028465	0.650277	0.000048	0.000003	0.321207
1	7425	ST-828 complex	33	39	30	82	113	77	17	0.047907	0.810917	0.001851	0.004866	0.134459
1	8480	not assigned	364	633	536	274	708	572	94	0.169992	0.332961	0.002747	0.002067	0.492233

SUPPLEMENTARY INFORMATION

Questionnaire for the case-control study

Information about your illness

First we would like to ask you about the symptoms caused by your *Campylobacter* infection.

1. **Which of the following symptoms of illness did you have?** More than one answer is possible. If the question does not apply to you, please answer with "No".

	Yes	No	Don't know
Loose and/or watery stools	☐	☐	☐
Diarrhoea (3 oder more unformed stools in 24 hours)	☐	☐	☐
Visible blood in stool	☐	☐	☐
Abdominal pain	☐	☐	☐
Nausea	☐	☐	☐
Vomiting	☐	☐	☐
Headache	☐	☐	☐
Fever (i.e., body temperature above 38,5°C)	☐	☐	☐
Other symptoms of illness	☐	☐	☐

↓

1.1 If Yes, which other symptoms did you have related to your *Campylobacter* infection?

____

2. **When did the diarrhoea begin? If you did not have diarrhoea, when did the first symptoms of illness begin?**
Please give the date as precisely as possible.

|_|_| Day |_|_| Month |_|_|_|_| Year — Don't know ☐

3. **On how many days in total did you have diarrhoea? If you did not have diarrhoea, how many days did the symptoms you decribed above last?**

|_|_| Days — Don't know ☐

3.1 Are the symptoms still ongoing? Yes ☐ No ☐ Don't know ☐

4. **Did you take an antibiotic because of the *Campylobacter* infection (for example, erythromycin, ciprofloxacin)?**

Yes ☐ No ☐ Don't know ☐

↓

4.1 If Yes, which one(s)?

____

5. **Did you have to stay away from work <u>because of the *Campylobacter* infection</u>?**

Yes ☐

No ☐ → **Please continue with question 6**

I am not employed. ☐ → **Please continue with question 6**

↓

5.1 If Yes, for how many working days?

|__|__| Working days

I am still unable to work ☐

6. **Were you treated in a hospital <u>because of the *Campylobacter* infection</u> (with at least one overnight stay)?**

Yes ☐

No ☐ → **Please continue with question 7.**

↓

6.1 If Yes, how long did you stay in the hospital?
Please report the number of nights that you spent in hospital.

|__|__| Nights

Don't know ☐

Travelling

Here we would like to know if you could have become infected with *Camplyobacter* during a trip to another country.

7. Have you travelled to another country <u>in the seven days before the onset of your illness</u>?

Yes ☐ No ☐ → **Please continue with question 8.**

↓

7.1 If Yes, which country/countries have you been to?

____

Contact with water

8. Did you go swimming/paddling <u>in the seven days before the onset of your illness?</u>

Yes ☐ No ☐ → **Please continue with question 9.**

Don't know ☐ → **Please continue with question 9.**

↓

8.1 If Yes, where did you go swimming or paddling?

More than one answer is possible. If the question does not apply to you, please answer with "No".

	Yes	No	Don't know
In a public swimming pool (outdoor or indoor)	☐	☐	☐
In a lake, river, stream	☐	☐	☐
In an ocean	☐	☐	☐
In a private swimming pool with chlorinated water	☐	☐	☐
In a pool/paddling pool with unchlorinated water	☐	☐	☐

Preparation and consumption of food

In the following section we would like to ask you about food items that you eat or drink as part of your normal diet as well as food items that you ate or drank in the seven days before the onset of your illness. It may be helpful to have a calendar at hand and look up special events or appointments you may have had in this time period.

9. Do you follow one of the following diets predominantly ?

Vegetarian or vegan	☐
Koscher (Jewish dietary laws)	☐
Halal (Islamic dietary laws)	☐
Special diet (e.g. macrobiotic diet; food combining diet)	☐ → **If Yes, please continue with question 9.1**
None of the listed diets/ no diet	☐
Don't know	☐

9.1 What kind of diet do you follow predominantly?

____

10. Do you eat meat, meat products, or sausage products?

Yes ☐ No ☐ → **Please continue with question 15.**

Meat, meat products and sausage products

11. **How often (on average) do you eat meat, meat products, or sausage products that are made from parts of the following animals?**
Please choose the answer that applies best to you.

	Several times per week	About once per week	About one to three times per month	Less than once per month	Never
Pig	☐	☐	☐	☐	☐
Cattle or calf	☐	☐	☐	☐	☐
Sheep or lamb	☐	☐	☐	☐	☐
Poultry (e.g., chicken, turkey)	☐	☐	☐	☐	☐
Game (e.g., boar, deer, rabbit, pheasant)	☐	☐	☐	☐	☐

12. **<u>In the seven days before the onset of your illness</u> did you eat the following kinds of meat or meat products? Please indicate also the state of the meat (e.g., raw or medium rare).**
More than one answer is possible. If the question does not apply to you, please answer with "No".

**As schnitzel, diced/sliced meat, roast, or steak etc.**	Raw	Partly raw or undercooked	Completely cooked	No	Don't know
Chicken	☐	☐	☐	☐	☐
Turkey	☐	☐	☐	☐	☐
Duck or goose	☐	☐	☐	☐	☐
Pork	☐	☐	☐	☐	☐
Beef or veal	☐	☐	☐	☐	☐
Mutton or lamb	☐	☐	☐	☐	☐
Game (e.g., boar, deer, rabbit, pheasant)	☐	☐	☐	☐	☐
**Ground/minced meat**	Raw	Partly raw or undercooked	Completely cooked	No	Don't know
Half and half (pork <u>and</u> beef)	☐	☐	☐	☐	☐
Pork (e.g. Mett/Hackepeter (=ground pork with spices), meat loaf)	☐	☐	☐	☐	☐
Beef (e.g. beef tatar)	☐	☐	☐	☐	☐
Poultry	☐	☐	☐	☐	☐
**Offal (e.g. liver, kidney)**	Raw	Partly raw or undercooked	Completely cooked	No	Don't know
Poultry	☐	☐	☐	☐	☐
Pig	☐	☐	☐	☐	☐
Cattle or calf	☐	☐	☐	☐	☐

13. **<u>In the seven days before the onset of your illness</u> did you eat <u>grilled</u> meat at home or at a friend's or a relative's home?**

Yes ☐ ↓

No ☐ → **Please continue with question 14.**

Don't know ☐ → **Please continue with question 14.**

13.1 What kind of meat did you eat? More than one answer is possible.
If the question does not apply to you, please answer with "No".

	Yes	No	Don't know
Sausage made from pork	☐	☐	☐
Sausage made from poultry meat	☐	☐	☐
Sausage made from beef or veal	☐	☐	☐
Chicken meat (e.g., chicken breast, chicken legs)	☐	☐	☐
Turkey meat (e.g., turkey steak, turkey breast)	☐	☐	☐
Pork (e.g., pork belly, cutlet, skewers)	☐	☐	☐
Beef or veal (e.g., beef steak, fillet)	☐	☐	☐
Mutton or lamb (e.g., steak, fillet)	☐	☐	☐
Ground meat (e.g., hamburger, cevapcici)	☐	☐	☐

14. **In the seven days before the onset of your illness did you eat the following sausage products/cold cuts?**

More than one answer is possible. If the question does not apply to you, please answer with "No".

	Yes	No	Don't know
Mettwurst	☐	☐	☐
Teewurst	☐	☐	☐
Salami or mini salami	☐	☐	☐
Cervelatwurst	☐	☐	☐
Sausage/cold cuts that contains poultry meat (chicken or turkey)	☐	☐	☐
Raw ham, smoked (e.g. Schwarzwälder, Holsteiner, Westfälischer Schinken)	☐	☐	☐

Other food items

15. **<u>In the seven days before the onset of your illness</u> did you eat one or more of the following food items?**
More than one answer is possible. If the question does not apply to you, please answer with "No".

	Yes	No	Don't know
Bagged lettuce or lettuce mix (ready-to-eat)	☐	☐	☐
Raw vegetables (e.g. tomato, cucumber, pepper, mushroom)	☐	☐	☐
Sprouts or seedlings (e.g. alfalfa, mung bean, soy bean, cress) or sprout salad	☐	☐	☐
Unpeeled fruit (e.g., apples, pears, nectarines)	☐	☐	☐
Fresh herbs (e.g. parsley, basil)	☐	☐	☐
Food items that contain raw eggs (e.g. tiramisu, home-made mayonnaise)	☐	☐	☐
Raw mussels (e.g. oysters)	☐	☐	☐

Milk products

16. **<u>In the seven days before the onset of your illness</u> did you drink raw milk or eat products made from unpasteurised milk?**
More than one answer is possible. If the question does not apply to you, please answer with "No".

Raw milk is untreated milk that was not heated above 40°C (unpasteurised). Raw milk can be purchased directly from a farmer („**milk from a farm**") or, specially labelled, in a store („**certified raw milk**").

	Yes	No	Don't know
Raw milk from a farm	☐	☐	☐
Certified raw milk	☐	☐	☐
Raw milk product (e.g., raw milk cheese)	☐	☐	☐

16.1. If you answered at least one question with "Yes", did you <u>always</u> heat the raw milk before you consumed it?

Yes ☐ No ☐ Don't know ☐

17. **<u>In the seven days before the onset of your illness</u> did you eat food items that were purchased directly on a farm or in a farm store?**

Yes ☐

No ☐ → **Please continue with question 18.**

Don't know ☐ → **Please continue with question 18.**

17.1 If "Yes", what kind(s) of food was that?

More than one answer is possible. If the question does not apply to you, please answer with "No.

	Yes	No	Don't know
Meat/meat products	☐	☐	☐
Vegetables/lettuce/herbs	☐	☐	☐
Fruit	☐	☐	☐
Milk/milk products	☐	☐	☐
Eggs	☐	☐	☐

18. **Does your <u>professional</u> work or the professional work of a person living in your household involve contact with raw meat, e.g. as a cook, in a butcher's, or a slaughterhouse?**

	Yes	No
My work	☐	☐
Work of another person living in the same household	☐	☐

18.1 If "Yes", what kind of profession do you (or the other person) have?

____

Preparation of food

19. **Which person in your household predominately prepares the food?**
Please give only one answer.

Me	☐
Another person	☐

↓

19.1 If you chose „Another person“, please let us know what your relationship with that person is (e.g. spouse).

Please do not tell us that person's name.

____

20. **In the seven days before the onset of your illness did you prepare raw meat in your household?** Please give only one answer.

Yes	☐	
No	☐	
Don't know	☐	
Raw meat is not prepared in my household.	☐	→ **Please continue with question 28**

21. **How often (on average) is poultry meat prepared in your household?**
Please give only one answer.

Several times per week	☐
About once per week	☐
About one to three times per month	☐
Less than once per month	☐
Never	☐ → **Please continue with question 23**
Don't know	☐

22. **In the seven days before the onset of your illness was one or more of the following poultry meat products prepared in your household?**
More than one answer is possible. If the question does not apply to you, please answer with "No".

**Fresh poultry meat (e.g. from the meat counter or a farmer's market)**	Yes	No	Don't know
Whole chicken	☐	☐	☐
Chicken meat (e.g., breast, leg, wing)	☐	☐	☐
Turkey meat (e.g., breast, leg, wing)	☐	☐	☐
Ground poultry meat	☐	☐	☐
Offal (e.g, liver, kidney) from chicken or turkey	☐	☐	☐
**Packaged meat (e.g. from the refrigerated counter)**	Yes	No	Don't know
Whole chicken	☐	☐	☐
Chicken meat (e.g. breast, leg, wing)	☐	☐	☐
Turkey meat (e.g. breast, leg, wing)	☐	☐	☐
Ground poultry meat	☐	☐	☐
Offal (e.g, liver, kidney) from chicken or turkey	☐	☐	☐
Ready-made poultry products (e.g., fried fillet strips)	☐	☐	☐
**Frozen poultry products**	Yes	No	Don't know
Whole chicken	☐	☐	☐
Chicken meat (e.g. breast, leg, wing)	☐	☐	☐
Turkey meat (e.g. breast, leg, wing)	☐	☐	☐
Ground poultry meat	☐	☐	☐
Offal (e.g, liver, kidney) from chicken or turkey	☐	☐	☐
Ready-made poultry products (e.g. chicken nuggets, breaded schnitzel)	☐	☐	☐

23. **How often (on average) is <u>pork</u> prepared in your household?**
Please give only one answer.

- Several times per week ☐
- About once per week ☐
- About one to three times per month ☐
- Less than once per month ☐
- Never ☐
- Don't know ☐

24. **How often (on average) is <u>beef or veal</u> prepared in your household?**
Please give only one answer.

- Several times per week ☐
- About once per week ☐
- About one to three times per month ☐
- Less than once per month ☐
- Never ☐
- Don't know ☐

25. **<u>In the seven days before the onset of your illness</u> did you prepare one or more of the following food items and raw meat together (for the same meal) ?**

More than one answer is possible. If the question does not apply to you, please answer with "No".

	Yes	No	Don't know
Lettuce	☐	☐	☐
Vegetables that were eaten <u>uncooked</u> (e.g. tomato, cucumber, pepper)	☐	☐	☐
Sprouts or seedlings (e.g. alfalfa, mung bean, soy bean sprouts, cress)	☐	☐	☐
Fresh herbs (e.g. parsley, basil)	☐	☐	☐
Fruit	☐	☐	☐

26. **Do you use separate utensils (e.g., cutting board, knife) for the preparation of raw meat and other food items (e.g., raw vegetables)?**

Always ☐ Most of the time ☐ Don't know ☐
Sometimes ☐
Never ☐

26.1 **If you anwered „Most of the time“, „Sometimes“ or „Never“, do you clean kitchen utensils (e.g, cutting board, knife) with hot water and soap <u>immediately after preparing raw meat</u>?**

Yes ☐ No ☐ Don't know ☐

27. **In your household are kitchen utensils that came into contact with raw meat cleaned in a dishwasher?**

Always ☐ Most of the time ☐ Don't know ☐
Sometimes ☐
Never ☐

Eating out

28. **How often (on average) do you eat out, e.g. in a restaurant, a canteen, at a friend's home?**
Please give only one answer.

- Several times per week ☐
- About once per week ☐
- About one to three times per month ☐
- Less than once per month ☐
- Never ☐

29. **<u>In the seven days before the onset of your illness</u> did you eat out, e.g. in a restaurant, canteen, at a friend's home, or eat take-out food?**

Yes ☐

No ☐ → **Please continue with question 31**

Don't know ☐ → **Please continue with question 31**

29.1 **If "Yes", where did you eat out or buy take-out food?**

More than one answer is possible.If the question does not apply to you, please answer "No".

	Yes	No	Don't know
At a bakery or a café	☐	☐	☐
At a restaurant	☐	☐	☐
At a fast-food chain restaurant	☐	☐	☐
At a food stall or similar (e.g., doner stall)	☐	☐	☐
At a canteen (e.g., at work, at university)	☐	☐	☐
In school/day care center	☐	☐	☐
In a private household (friend(s)/relative(s))	☐	☐	☐
In a hospital/nursery home	☐	☐	☐

30. **When eating out <u>in the seven days before the onset of your illness</u> did you eat one or more of the following food items?**
More than one answer is possible.If the question does not apply to you, please answer "No".

	Yes	No	Don't know
Sandwich, baguette	☐	☐	☐
Asian dish with chicken (e.g., fried noodles)	☐	☐	☐
Duck meat	☐	☐	☐
Chicken doner	☐	☐	☐
Grilled chicken	☐	☐	☐
Chicken schnitzel or poultry meat strips	☐	☐	☐
Chicken burger/chicken nuggets/chicken wings	☐	☐	☐
Hamburger or other burgers with beef or pork (on a bun)	☐	☐	☐
Beef or veal steak	☐	☐	☐
Bratwurst/Currywurst	☐	☐	☐
Hamburger meat from pork (e.g., Bulette/Frikadelle) (without bun)	☐	☐	☐
Eggs (e.g., on a salad, on a sandwich)	☐	☐	☐
Lettuce or lettuce mix	☐	☐	☐
Sprouts or seedlings (e.g., alfalfa, mung bean, or soy bean sprouts, cress) or sprout salad	☐	☐	☐
Precut fruit or berries	☐	☐	☐
Raw mussels (e.g., oysters)	☐	☐	☐
Sushi	☐	☐	☐

Animal contact

31. In the seven days before the onset of your illness did you have contact with animals (pets, farm animals, wild animals), that is, did you touch the animals or come into contact with their faeces?

Yes ☐ ↓

No ☐ → **Please continue with question 32**

Don't know ☐ → **Please continue with question 32**

31.1 In the seven days before the onset of your illness did you touch one or more of the following animals (or the animals' faeces) in one of the listed locations?

More than one answer is possible.If the question does not apply to you, please answer "No".

**PETS**	Own household/ farm	Zoo, petting zoo, game reserve	On a visit (e.g., to farm, friends)	Other location	No	Don't know
Dogs	☐	☐	☐	☐	☐	☐
Cats	☐	☐	☐	☐	☐	☐
Rodents (e.g. hamsters, guinea pigs, rabbits, rats)	☐	☐	☐	☐	☐	☐
Birds (e.g. parrots, budgerigars)	☐	☐	☐	☐	☐	☐
Reptiles (e.g. turtles, snakes, lizards)	☐	☐	☐	☐	☐	☐
**FARM ANIMALS**	Own household/ farm	Zoo, petting zoo, game reserve	On a visit (e.g., to farm, friends)	Other location	No	Don't know
Cattle or calves	☐	☐	☐	☐	☐	☐
Pigs	☐	☐	☐	☐	☐	☐
Sheep	☐	☐	☐	☐	☐	☐
Goats	☐	☐	☐	☐	☐	☐
Horses	☐	☐	☐	☐	☐	☐
Chickens	☐	☐	☐	☐	☐	☐
Ducks/Geese	☐	☐	☐	☐	☐	☐
**WILD ANIMALS**	Own household/ farm	Zoo, petting zoo, game reserve	On a visit (e.g., to farm, friends)	Other location	No	Don't know
Boars	☐	☐	☐	☐	☐	☐
Deers	☐	☐	☐	☐	☐	☐
Wild birds (e.g., pigeons, sparrows)	☐	☐	☐	☐	☐	☐

32.	In the seven days before the onset of your illness did you touch sand from a sandbox or on a playground (e.g., in children's day care centre)?

Yes ☐ No ☐ Don't know ☐

33.	Does your professional work or the professional work of a person living in your household involve animal contact, e.g., as a farmer, veterinarian?

	Yes	No
My work	☐	☐
Work of another person living in the same household	☐	☐

33.1 If "Yes", what kind of profession do you (or the other person) have?

____

Contact persons

34.	How many persons are living in your household, including yourself?

|_|_| Persons

35.	Are children living in your household that…

	Yes	No
ever wear diapers?	☐	☐
attend kindergarten/day care centre?	☐	☐

36. Does your <u>professional</u> work or the professional work of a person living in your household involve contact with children under six years of age, e.g., in a kindergarten?

	Yes	No
My work	☐	☐
Work of another person living in the same household	☐	☐

36.1 If "Yes", what kind of profession do you (or the other person) have?

____

37. Do you know of anyone else who had diarrhoea at the same time as you (or in the two weeks before or after the onset of your illness) ?

Yes ☐

No ☐ → **Please continue with question 38**

Don't know ☐ → **Please continue with question 38**

37.1 If "Yes", who is this person/are these persons?

Please indicate, if these persons developed symptoms "at the same time", "earlier" or "later", compared to you. More than one answer is possible.

	Same time (+/- 3 days)	Earlier (more than 3 days)	Later (more than 3 days)
Person living in the same household	☐	☐	☐
Colleague at work or fellow student	☐	☐	☐
Friend, acquaintance, relative	☐	☐	☐
Other person	☐	☐	☐

37.2 If you chose „Other person“, please let us know what your relationship with that person is. Please <u>do not</u> tell us that person's name.

____

Medication

38. **In the <u>four weeks before the onset of your *Campylobacter* infection</u> did you take antibiotics (e.g., penicillin, amoxicillin, doxycyclin) because of another illness?**

Yes ☐ No ☐ Don't know ☐

↓

38.1 If "Yes", which one(s)?

____

39. **In the <u>four weeks before the onset of your *Campylobacter* infection</u> did you take gastric acid inhibitors, i.e., medication that reduces gastric acid (e.g., Omeprazol, Pantoprazol, Ranitidin®, Nexium®), because of other health problems?**

Yes ☐ No ☐ Don't know ☐

↓

39.1 If "Yes", which one(s)?

____

40. **In the <u>four weeks before the onset of your *Campylobacter* infection</u> did you take medication or receive treatment (e.g. chemotherapy) that weakens the immune system?**

Yes ☐ No ☐ Don't know ☐

↓

40.1 If "Yes", which one(s)?

____

41. **Have you ever been diagnosed with one or more of the following diseases?**
More than one answer is possible.If the question does not apply to you, please answer "No".

	Yes	No
Diabetes mellitus	☐	☐
Chronic inflammatory intestinal disease (e.g. Crohn's disease)	☐	☐
Stomach ulcer	☐	☐
Cancer	☐	☐

42. **Have you ever been diagnosed with a *Campylobacter* infection before?**

Yes ☐ No ☐ Don't know ☐

Personal information

43. Please let us know your month and year of birth:

|_|_| |_|_|_|_|

Month Year

44. Please indicate your sex:

female ☐ male ☐

45. What is the zip code of your home address?

|_|_|_|_|_|

46. Which country were you born in?

In Germany ☐

In another country ☐ in : ______________________

47. What citizenship do you have?

German ☐ Other citizenship ☐

Which one? ____________________

48. Since when have you predominantly lived in Germany?

Since my birth ☐ or since (year) |_|_|_|_|

49. What is your professional training?

Please indicate the highest degree only.

Still in professional training (trainee, university student, high school student)	☐
No professional degree (and not in training)	☐
Apprenticeship qualification ("Lehre" (dual vocational training)), commercial college, technical college or similar	☐
Advanced technical college, engineering college or similar	☐
University	☐
Other degree	☐

Please enter today's date:

|__|__| |__|__| |__|__|__|__|

Day Month Year

Would you like to comment on the study or this questionnaire?

Please write down your comments here.

Written Consent

Hereby I, ______________________________ ,
(Last name, first name)

living in ______________________________
(Street, street number)

______________________________ ,
(Zip code, town)

delare that I have been informed in writing in an accompanying letter about the "Population based study on risk factors and molecular epidemiology of *Campylobacter* infections in humans". I agree to participate in this scientific study. I agree that data obtained as part of this study will be anonymised and saved electronically and that the anonymised study results will be published. I have been informed that I can withdraw my consent at any time without giving reasons and without disadvantages for me, and that I can withdraw my permission for the processing of my data.

If you would like to withdraw your permission, please contact the Robert Koch Institute at 030-18754-3360.

I agree that the *Campylobacter* bacterial sample from my stool sample can be analysed further.

No ☐ Yes ☐

______________________________ ______________________________
(Place, Date) Signiture of participant

May we contact you again during this study if we have questions?

Yes ☐ No ☐

If you agree to be contacted again, what is the best way to reach you?

Telephone number or email address:

____

Please put the completed questionnaire in the stamped addressed envelope provided.

This page will be separated from the rest of the questionnaire and stored separately at the Robert Koch Institute.
